# Structural homology of HHV-6B epitopes as candidates for molecular mimicry triggers of the onset type one diabetes mellitus

**DOI:** 10.1093/oxfimm/iqag011

**Published:** 2026-07-03

**Authors:** McKay Jones, Julio C Facelli

**Affiliations:** Utah Valley University, Orem, UT, United States; Department of Biomedical Informatics, Spencer Fox Eccles School of Medicine, The University of UT, Salt Lake City, UT, United States; Department of Biomedical Informatics, Spencer Fox Eccles School of Medicine, The University of UT, Salt Lake City, UT, United States

**Keywords:** HHV-6B, molecular mimicry, T1DM, structural homology, protein structure prediction, autoinmunity, roseola

## Abstract

**Objectives:**

To study whether HHV-6B can act as a molecular mimic to trigger onset of Type One Diabetes Mellitus (T1DM) by assessing structural and binding similarities between HHV-6B derived epitopes and T1DM autoantigen derived epitopes.

**Methods:**

Epitope peptide structures and their interactions with T1DM autoantigens were modeled using Boltz-2, a state-of-the-art artificial intelligence protein structure prediction method.

**Results:**

Several HHV-6B epitopes studied here demonstrated high structural alignment with T1DM antigen epitopes, but even when structural homology was lacking, in multiple cases the HHV-6B and T1DM epitopes fit into the same binding region of the corresponding HLA molecules, suggesting a plausible structural basis for T cell cross-reactivity. These findings support the hypothesis that HHV-6B may act as a molecular mimic contributing to autoimmune responses in individuals genetically susceptible to T1DM.

**Conclusion:**

This study demonstrates that structural modeling is a useful tool for identifying potential mimicry candidates that sequence-based methods may not find, underscoring the importance of integrating structure-based modeling, including docking, into molecular mimicry prediction pipelines for identifying potential epitopes for *in vitro* studies.

## Introduction

Although the etiology of type one diabetes mellitus (T1DM) is not fully understood, it likely involves an interplay of genetic, environmental, dietary, and viral triggers [1]. In a previous study, Mistry *et al*. [2] found that individuals in The Environmental Determinants of Diabetes in the Young (TEDDY) study cohort [3] who experienced at 12 months of age a single episode of exanthema subitem, commonly known as roseola, and for which the HHV-6B virus is generally considered the primary causative agent, had a 4.49-fold increased risk of developing T1DM. This finding motivates the hypothesis that molecular mimicry, which arises when foreign peptides share similarities with self-antigens, triggering a cross-reactive immune response, could be a contributing mechanism behind this substantial increase. This hypothesis is further supported by the increasing body of evidence that implicates molecular mimicry as a contributor to autoimmune diseases (ADs), including type 1 diabetes mellitus (T1DM) [4–7]. Paradoxically, no HHV-6B epitopes are currently (as 25 August 2025) listed in the Immune Epitope Database (IEDB) [8]. Bach *et al*. [9] reported strong reactivity of GAD65-specific T cells toward several epitope candidates, including one from HHV-6B. These findings may be explained because the results of the Bach study are not included in the IEDB. Moreover, the GAD65 (GAD: 248–259 and GAD: 246–257) and HHV-6B U2 epitopes in the study exhibit low sequence homology with known autoimmune targets [10]. Nevertheless, structural homology between GAD65 and infectious peptides (such as HHV-6B), even without amino acid similarities, could still permit a cross-reactive response [11]. An additional source of HHV-6B epitope candidates for molecular mimicry can be found in the recent Suleman *et al*. study designing a vaccine against HHV-6A [12], where we can identify several candidate epitopes that show a great sequence homology with their corresponding sequences in HHV-6B (see Supplementary Material Figure S1, available as supplementary material at *OXFIMM Journal* online).

The purpose of this study is to computationally evaluate structural homology and binding similarity between the HHV-6B epitope identified in the Bach’s study and the HHV-6B epitopes homologous to those reported by Suleman *et al*. for HHV-6A [12].

## Methods

The study by Bach *et al*. [9] identified candidate mimicry peptides by screening protein databases using epitope motifs derived from GAD65 sequences and performing the corresponding immunological studies. Of interest here are three epitopes that show strong reactivity with a panel of homozygous DRB5*01:01 B cells. Using the numbering from Table 2 in [9] these three epitopes, given in Table 1, are: GAD: 248–259 (epitope 3), GAD: 246–257 (epitope 4), and HHV-6 U2 (epitope 7).

**Table 1:** Bach epitopes considered here.

Identification #	Epitope name	Sequence
3	GAD65 (248–259)	MYAMMIARFKMF
4	GAD65 (246–257)	SNMYAMMIARFK
7	Human herpesvirus-6 U2	GGVAVVIGRFFG

**Table 2:** Epitopes from Suleman *et al*. showing sequential matching with T1DM antigen epitopes.

E-T1D ID	E-T1D	E-HHV6	E-T1D MHC molecule	RMSD	E-T1D antigen name
163076	SDPKQ	TDPKQ	HLA-A*01:01	0.98	Nardilysin
185874	LTDPVTIC	LTDPKQTC	HLA-B*44:02	No match	Genome polyprotein
430465	TDAGTGRPY	TDINFKAPY	HLA-A*01:01	No match	Rho GTPase-activating protein 27
431152	DIHGNVLQY	DILYVQLQY	HLA-A*01:01	No match	3'(2')5'-bisphosphate nucelotidase 1
431175	LTDPM	LTDPK	HLA-A*01:01	0.82	Deoxyribonuclease TATDN1
431177	LTDPSSPTI	LTDPKQTCI	HLA-A*01:01	No match	Spindle and kinetochore-associated protein 3
431594	TDFYQTSY	TDPKQTCI	HLA-A*01:01	No match	Protein YIPF5
448259	SHTSVGN	SHCKNGN	HLA-B*38:01	No Match	Glucose-6-phosphate exchanger SLC37A1
449222	THMTAI	TAMTAI	HLA-B*15:10	No Match	ATP-citrate synthase
468440	LTDDQAKY	LTTTNIKY	HLA-A*01:01	No match	S-adenosylhomocysteine hydrolase-like protein 1
482558	EVVTQQY	EVRQMQY	HLA-B*18:01	No match	Thrombopoietin receptor
488701	TTNIQ	TTNIK	HLA-B7	No match	Thrombopoietin receptor
541490	DIKARALQ	DILYVQLQ	HLA-B*08:01	0.23	Polycomb group protein ASXL1
562998	ILPIMNQY	ILYVQLQY	HLA-A*25:01	No match	Mitochondrial Rho GTPase 1
563936	VVDEHTGQY	VVEVRQMQY	HLA-A*26:01	12.38	Jouberin
571479	DIRKKRLQ	DILYVQLQ	HLA-A*08:01	0.46	Centromere protein H
571480	DIRQKA	DINFKA	HLA-B*08:01	No match	DNA polymerase alpha catalytic subunit
573185	EVLLPQY	EVRQMQY	HLA-B*18:01	No match	G1/S-specific cyclin-E1
602576	ILPGNLQSW	ILYVQLQYL	HLA-B*57:01	No match	Zinc finger protein with KRAB and SCAN domains 4
620896	MDINF	TDINF	HLA-A*23:01	No match	Unconventional myosin-Id
625331	SIFTVK	NIFTVQ	HLA-B*38:02	1.23	Kinesin-like protein KIF20B
771155	DIRKKAA	DINFKAP	HLA class I	No match	U5 small nuclear ribonucleoprotein 40 kDa protein
890415	ESYLKN	ESHCKN	HLA-B*08:01	3.27	DNA replication complex GINS protein SLD5
935014	TAEALAAF	TAMTAIAF	HLA-A*25:01	0.32	Nucleolar protein 58
935131	VVEVAGL	VVEVRQM	HLA-A*25:01	No match	Sperm-associated antigen 7

The RMSD value corresponds to the calculated difference between the boltz-2 predicted structures (see text for details).

Suleman *et al*. [12] built a computationally designed multi-epitope subunit vaccine against HHV-6A, using nine immunodominant epitopes from Glycoproteins B, H, and Q of the HHV-6A proteome, which show great sequence similarity with those regions of HHV-6B (see Supplementary Material Figure S1, available as supplementary material at *OXFIMM Journal* online). therefore making these epitopes also reasonable candidates for our study.

The HHV-6B and HHV-6A epitopes only bind to major histocompatibility complex I (MHC-I) molecules [13] so T1DM epitopes were filtered to only include *Homo sapiens*, and MHC-I associated epitopes. A set of 8,238 T1DM epitopes were downloaded from the IEDB [8] on June 23, 2025. We searched for sequence homology between this set of epitopes and each of HHV-6B epitopes found by homology with those of HHV-6A to identify potential pairs of epitopes that could be candidates for triggering molecular mimicry. Amino acid sequence comparisons between the two datasets were done using the BLASTp algorithm with default parameters at the Utah Center for High Performance computing (https://www.chpc.utah.edu/) following the method previously published [14]. This yielded 25 unique pairs characterized by matching sequences of at least 5 amino acids with no gaps in the aligned regions. These epitope pairs are listed in Table 2 and were considered here as potential triggers of autoimmunity.

The HLA molecules sequences were obtained from https://www.ebi.ac.uk/ipd/ [15].

The 50 epitopes from the 25 candidate pairs found by sequence homology from the Suleman studies were labeled by the identifiers entered in Table S1, available as supplementary material at *OXFIMM Journal* online as E-T1D ID, which corresponds to the ID in IEDB for the T1DM epitope in the pair and those three from Bach’s paper. Structural modeling of these 53 epitopes was performed with Boltz-2 (v.2.2.0). Boltz-2 is an open-source deep learning method that predicts biomolecular structures and binding sites [16] with great accuracy. Boltz-2 requires protein inputs of at least 10 amino acids, and many of the epitopes considered here (Tables 1 and 2) did not meet this requirement, so each epitope sequence was expanded symmetrically using the full protein sequences from UniProt (https://www.uniprot.org/). The extended sequences are given in Table S1 of the Supplementary Material, available as supplementary material at *OXFIMM Journal* online and the full FASTA files used in the modeling are available at https://doi.org/10.5281/zenodo.17832559.

All Boltz-2 calculations were performed at the Utah Center for High Performance Computing. Boltz-2 generates four structural models per input, but all results presented here are based on the first model, which had the highest accuracy. ChimeraX was used to visualize the calculated structures and to calculate structural and electrostatic similarity. RMSD values between structures were computed using the “matchmaker” tool in ChimeraX, which superimposes protein structures through pairwise sequence alignment. Electrostatic surface potentials were calculated using ChimeraX’s Coulombic Surface Coloring tool [17] and the palette options were colored as follows: blue is defined to be negative, red is defined to be positive, and white is defined to be neutral. All the Boltz-2 results are available at https://doi.org/10.5281/zenodo.17832559.

## Results

In all cases selected for analysis, model_0 from Boltz-2 consistently exhibited the highest confidence scores, and these models were used in subsequent analyses. The confidence scores for all the calculations are given in the Figs and compiled in Table S2 of the Supplementary Material, available as supplementary material at *OXFIMM Journal* online. The average confidence score is 0.74 for the structures of the isolated epitopes and 0.87 for the structures of the epitopes bound to the HLA molecules. These values are within the range of good Boltz-2 predictions [16] and in Table S1 there is only one bad score below the threshold of 0.6. This supports the use of Boltz-2 for exploratory computational studies of molecular mimicry.

**Figure 1:**
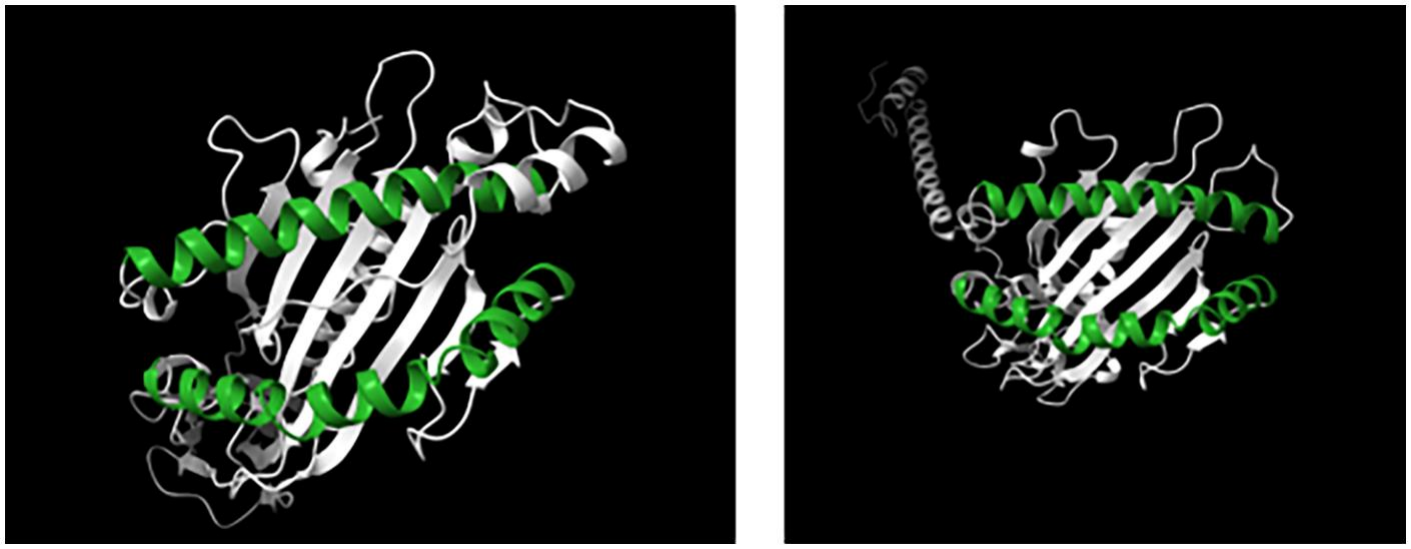
Boltz-2 structures of HLA-A*01:01 (left) and HLA-B*08:08 (right) showing the binding pocket provided by the sequences (in green) between PRO-81 to GLY-107 and MET- 162 to ASN-198 for HLA-A*01:01 and PRO-81 to TYR-108 and THR-162 to ASN-198 for HLA-B*09:01.

In this study we considered three classes of HLA molecules HLA-A*, HLA-B* and HLA-DRB5*. The predicted structures of the HLA molecules show high structural similarity between all the molecules in each class. For the HLA-A molecules the RMSD between the structures of HLA-A*01:01 and HLA-A*08:01, HLA-A*23:01, HLA-A*25:01 and HLA-A*26:01 are 0.62 Å, 0.57 Å, 0.58 Å and 0.49 Å, respectively. For the HLA-B molecules the RMSD between the structures HLA-B*08:01 and HLA-B*15:01, HLA-B*18:01, HLA-B*38:01, HLA-B*38:02, HLA-B*44:02 and HLA-B*57:01 are 0.78 Å, 0.91 Å, 0.89 Å, 0.92 Å, 0.80 Å and 0.50 Å, respectively. The structures of both HLA-A and HLA-B molecules show also a similar core structure depicting a clear pocket (see Fig. 1) between the coil structures observed for the sequences PRO-81 to GLY-107 and MET-162 to ASN-198 for HLA-A*01:01 and PRO-81 to TYR-108 and THR-162 to ASN-198 for HLA-B*09:01.

The structure of HLA-DRB5*01:01 is quite different and does not exhibit the pocket discussed above but instead shows a region of parallel β-sheets (GLY-38 to PHE-47) in which the HHV-6B epitopes bind (see below). For further details see Supplementary Material, available as supplementary material at *OXFIMM Journal* online.

**Figure 2:**
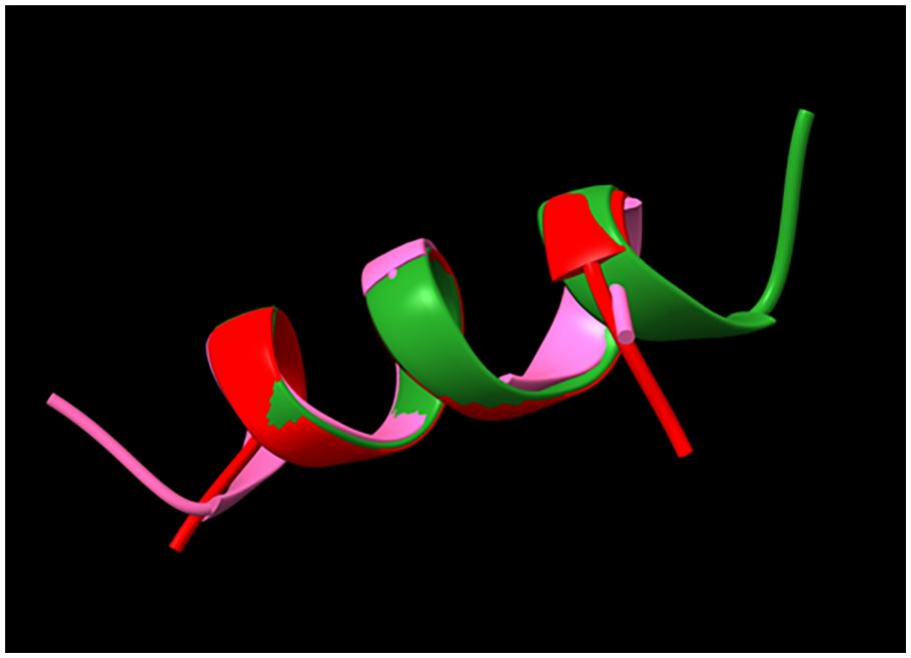
Comparison of the boltz-2 predicted structures of the isolated epitopes GAD65(248–259) (green) and GAD65(246–257) (pink) with the HHV-6 (red) epitope. Boltz-2 confidence scores for these isolated epitope structures are 0.78, 0.79, and 0.83.

**Figure 3:**
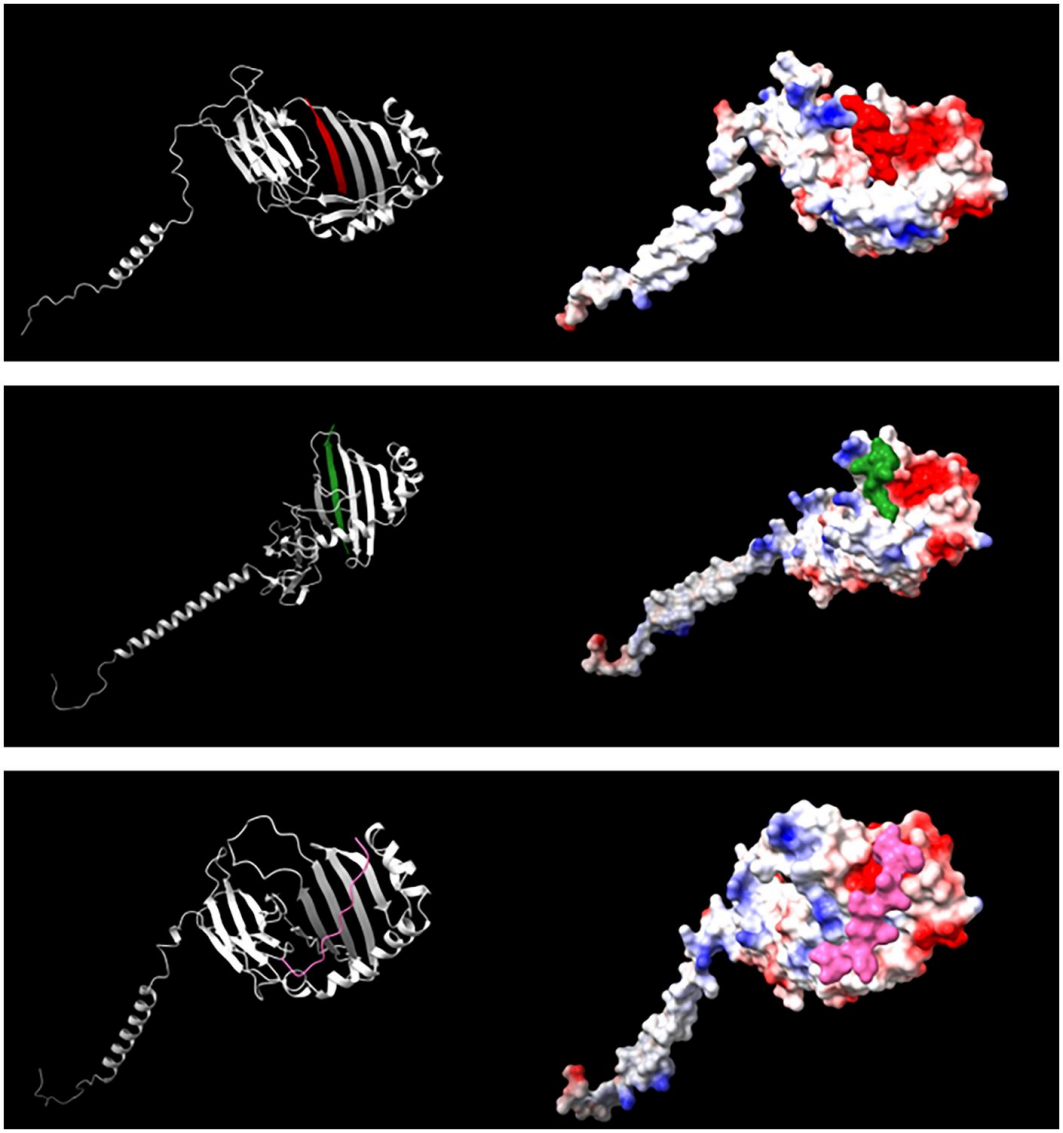
Top to bottom conformations of epitopes GAD65(248–259) (green), GAD65(246–257) (pink), and HHV-6B (red) bound to HLA-DRB5*01-01 (white). for the predicted structure of these complexes, boltz-2 confidence scores were 0.80, 0.85 and 0.83. The structures on the right in each panel correspond to the calculated complexes, potential of the complexes., where it can be observed the very similar bonding site for GAD65(248–259) and HHV-6B epitopes.

Superposition of the structures of the two GAD65 protein epitopes and Bach’s HHV-6B epitope, are shown in Fig. 2 and Table 3, revealed a good structural match with RMSD values of 0.254 Å and 0.553 Å, respectively. These isolated structures share a secondary coil, as depicted in Fig. 2. Figure 3 shows the conformations of epitopes GAD65(248–259) (green), GAD65(246–257) (pink), and HHV-6B (red) bound to HLA-DRB5*01:01 (white). It is observed that all the epitopes change folding upon binding to HLA-DRB5*01:01, GAD65(248–259) and HHV-6B epitopes adopt a β-sheet conformation and bind to the same region of HLA-DRB5*01:01, while GAD65(246–257) adopt a disordered configuration binding into a different region. The similar binding of the GAD65(248–259) and HHV-6B epitopes to the HLA-DRB5*01:01 molecule is a strong indication that HHV-6B can induce molecular mimicry, triggering the onset of T1DM by this mechanism.

**Table 3:** RMSD Calculations between the GAD65 protein epitopes and the HHV-6 epitope.

Epitope #1	Epitope #2	RMSD (Å)	Amino Acid Pairs
4	3	0.442	10
7	3	0.254	10
7	4	0.553	11

The results obtained for the epitopes derived from the Suleman’s work [12] are summarized in Table 4 and depicted in Figure S3 of the Supplementary Material, available as supplementary material at *OXFIMM Journal* online. The structural similarity of the isolated epitope pairs is relatively low, with only pairs corresponding to the index pairs 163076, 431175, 541490, 57149, and 935131 showing a match with a RMSD below 1 Å. The results for the epitopes bound to the corresponding HLA molecules are quite different, Table 4 and Figure S2 clearly show that most epitopes acquired an extended configuration, even when they exhibit a coiled structure when isolated. In the majority of cases both epitopes bind to the pocket discussed above (see Fig. 1) between the coil structures observed for the sequences between PRO-81 to GLY-107 and MET-162 to ASN-198 for HLA-A*01:01 and PRO-81 to TYR-108 and THR-162 to ASN-198 for HLA-B*09:01, which in Table 4 we denominate as site A. This is the case of pairs, 163076, 185874, 431175, 431177, 431594, 468440, 482558, 541490, 562998, 563936, 571479, 571480, 573185, 602576, 620896, 625331, 890415, 935014, which represent most of the pairs considered here. These results show that according to the Boltz-2 predictions, there are 18 T1DM/HHV-6B epitope pairs that have similar binding characteristics and represent candidates for molecular mimicry by HHV-6B infections. The results of the analysis of the binding sites using the calculated electrostatic potentials are presented in Figure S4 of the Supplementary Material, available as supplementary material at *OXFIMM Journal* online fully support the findings discussed above and provide intuitive depictions of the potential for molecular mimicry of the T1DM/HHV-6B epitope pairs listed above.

**Table 4:** RMS Between boltz-2 predicted HHV-6 and T1DM epitope structures and predicted binding sites to HLA molecules.

Epitope pair index	HHV-6/T1DM epitope RMSD (in Å)	HHV-6 and T1DM epitope binding site
163076	0.98	Both epitomes are binding to HLA site A
185874	No match	Both epitomes are binding to HLA site A
430465	No match	Different binding sites for both epitopes
431152	No match	Different binding sites for both epitopes
431175	0.82	Both epitomes are binding to HLA site A
431177	No match	Both epitomes are binding to HLA site A
431594	No match	Both epitomes are binding to HLA site A
448259	No match	Different binding sites for both epitopes
449222	No match	Different binding sites for both epitopes
468440	No match	Both epitomes are binding to HLA site A
482558	No match	Both epitomes are binding to HLA site A
541490	0.23	Both epitomes are binding to HLA site A
562998	No match	Both epitomes are binding to HLA site A
563936	12.38	Both epitomes are binding to HLA site A
571479	0.46	Both epitomes are binding to HLA site A
571480	No match	Both epitomes are binding to HLA site A
573185	No match	Both epitomes are binding to HLA site A
602576	No match	Both epitomes are binding to HLA site A
620896	No match	Both epitomes are binding to HLA site A
625331	1.23	Both epitomes are binding to HLA site A
890415	3.27	Both epitomes are binding to HLA site A
935014	0.32	Both epitomes are binding to HLA site A
935131	No match	Both epitomes are binding to the same site but the site for HLA-A-25 is different than site A

## Discussion

This study builds on the findings of Bach *et al*. [9] by demonstrating that peptides with low sequence similarity can nonetheless exhibit significant structural and electrostatic homology, resulting in good candidates for molecular mimicry. Using Boltz-2 and ChimeraX, we show that GAD65-derived and HHV-6B epitopes share similar electrostatic surface potentials, demonstrating that these epitopes may bind with comparable affinity to HLA-DRB5*01:01, a class II MHC allele previously linked to T1DM onset [9, 18]. Conversely, the results from the analysis of the epitopes derived from those from the Suleman work [12] show that in many cases epitopes showing significant sequence homology may not exhibit structural homology when considered as isolated entities but show also significant binding similarities when modeled with corresponding HLA molecules.

In this study we identified 19 potential HHV-6B epitopes that have potential to trigger T1DM by molecular mimicry. These findings are consistent with clinical results derived from the TEDDY cohort, [19] which reported a 4.49-fold increased risk for T1DM following roseola infection in infancy [2, 10].

The study also demonstrates that peptides lacking sequence homology can still exhibit significant structural and electrostatic biding similarity. These features may enable molecular mimicry, contributing to the onset of autoimmune diseases without significant sequence homology [9]. On the other hand, the results also indicate that sequence homology does not always lead to structural homology and that the lack of structural homology for isolated epitopes may not be equated to lack of binding similarities.

## Conclusion

Using novel bioinformatics tools, this study identifies multiple epitope candidates that could explain the previous clinical findings [2] on the strong association between roseola infections and the onset of T1DM presumably by HHV-6B molecular mimicry. While previous studies have shown that investigating sequence homology is an efficient and less computationally expensive approach for identifying epitopes with potential molecular mimicry capacity [9, 10], this work highlights that structural modeling, including binding calculations, is essential for uncovering molecular mimicry candidates that sequence-based methods may not find. These findings emphasize the importance of integrating structure-based modeling into molecular mimicry pipelines to identify good epitope candidates for *in vitro* studies. While in silico studies cannot replace *in vitro* validation large scale binding affinity may become increasingly feasible when using state-of-the-art AI-based protein molecular modeling tools like Boltz-2 providing good candidates for conclusive testing using *in vitro* experiments.

## Supplementary Material

iqag011_Supplementary_Data

## Data Availability

All the Boltz-2 results are available at https://doi.org/10.5281/zenodo.17832559
