# Supplementary material for: Structural homology of HHV-6B epitopes as candidates for molecular mimicry triggers of the onset type one diabetes mellitus
Source: Oxf Open Immunol. 2026 Jul 3;7(1):iqag011. doi: 10.1093/oxfimm/iqag011 (PMC13350986; doi:10.1093/oxfimm/iqag011)

**Supplementary Material**

**Table S1:** Extended Epitopes used in the Boltz-2 calculations.

| **E-T1D ID** | **E-T1D** | **E-T1D (EXT)** | **E-HHV6** | **E-HHV6 (EXT)** | **E-T1D MHC Molecule** | **E-T1D Antigen Name** | | **E-T1D Antigen UniProt ID** |
| --- | --- | --- | --- | --- | --- | --- | --- | --- |
| 163076 | SDPKQ | KSPSDPKQYRY | TDPKQ | LNLTDPKQTCI | HLA-A*01:01 | Nardilysin | | O43847 |
| 185874 | LTDPVTIC | YLTDPVTICC | LTDPKQTC | LNLTDPKQTCIK | HLA-B*44:02 | Genome polyprotein | | P17763 |
| 430465 | TDAGTGRPY | HTDAGTGRPYY | TDINFKAPY | TTDINFKAPYR | HLA-A*01:01 | Rho GTPase-activating protein 27 | | Q6ZUM4 |
| 431152 | DIHGNVLQY | TDIHGNVLQYH | DILYVQLQY | HDILYVQLQYL | HLA-A*01:01 | 3'(2')5'-bisphosphate nucelotidase 1 | | O95861 |
| 431175 | LTDPM | GINLTDPMFRG | LTDPK | SLNLTDPKQTC | HLA-A*01:01 | Deoxyribonuclease TATDN1 | | Q6P1N9 |
| 431177 | LTDPSSPTI | NLTDPSSPTIS | LTDPKQTCI | NLTDPKQTCIK | HLA-A*01:01 | Spindle and kinetochore-associated protein 3 | | Q8IX90 |
| 431594 | TDFYQTSY | NTDFYQTSYS | TDPKQTCI | LTDPKQTCIK | HLA-A*01:01 | Protein YIPF5 | | Q969M3 |
| 448259 | SHTSVGN | WNSHTSVGNIL | SHCKNGN | DESHCKNGNSE | HLA-B*38:01 | Glucose-6-phosphate exchanger SLC37A1 | | P57057 |
| 449222 | THMTAI | TETHMTAIVG | TAMTAI | PLTAMTAIAF | HLA-B*15:10 | ATP-citrate synthase | | P53396 |
| 468440 | LTDDQAKY | ELTDDQAKYL | LTTTNIKY | NLTTTNIKYD | HLA-A*01:01 | S-adenosylhomocysteine hydrolase-like protein 1 | | O43865 |
| 482558 | EVVTQQY | WNEVVTQQYLFD | EVRQMQY | VVEVRQMQYDN | HLA-B*18:01 | Thrombopoietin receptor | | P40238 |
| 488701 | TTNIQ | LVYTTNIQELN | TTNIK | KLTTTNIKYDI | HLA-B7 | Thrombopoietin receptor | | P40238 |
| 541490 | DIKARALQ | ADIKARALQV | DILYVQLQ | HDILYVQLQY | HLA-B*08:01 | Q8IXJ9 |  |  |
| 562998 | ILPIMNQY | TILPIMNQYT | ILYVQLQY | DILYVQLQYL | HLA-A*25:01 | Mitochondrial Rho GTPase 1 | | Q8IXI2 |
| 563936 | VVDEHTGQY | HVVDEHTGQYV | VVEVRQMQY | RVVEVRQMQYD | HLA-A*26:01 | Jouberin | | Q8N157 |
| 571479 | DIRKKRLQ | LDIRKKRLQL | DILYVQLQ | HDILYVQLQY | HLA-A*08:01 | Centromere protein H | | Q9H3R5 |
| 571480 | DIRQKA | QYDIRQKALK | DINFKA | TTDINFKAPY | HLA-B*08:01 | DNA polymerase alpha catalytic subunit | | P09884 |
| 573185 | EVLLPQY | LHEVLLPQYPQ | EVRQMQY | VVEVRQMQYDN | HLA-B*18:01 | G1/S-specific cyclin-E1 | | P24864 |
| 602576 | ILPGNLQSW | TILPGNLQSWV | ILYVQLQYL | DILYVQLQYLY | HLA-B*57:01 | Zinc finger protein with KRAB and SCAN domains 4 | | Q969J2 |
| 620896 | MDINF | GKYMDINFDFK | TDINF | GNTTDINFKAP | HLA-A*23:01 | Unconventional myosin-Id | | O94832 |
| 625331 | SIFTVK | SHSIFTVKIL | NIFTVQ | VNNIFTVQAR | HLA-B*38:02 | Kinesin-like protein KIF20B | | Q96Q89 |
| 771155 | DIRKKAA | LWDIRKKAAIQ | DINFKAP | TTDINFKAPYR | HLA class I | U5 small nuclear ribonucleoprotein 40 kDa protein | | Q96DI7 |
| 890415 | ESYLKN | NTESYLKNVA | ESHCKN | SDESHCKNGN | HLA-B*08:01 | DNA replication complex GINS protein SLD5 | | Q9BRT9 |
| 935014 | TAEALAAF | DTAEALAAFT | TAMTAIAF | LTAMTAIAFC | HLA-A*25:01 | Nucleolar protein 58 | | Q9Y2X3 |

**Table S2:** Boltz-2 confidence scores for all the structures presented in Figures S2 and S3. Boltz‑2 scores are considered good for 0.8 to 1.0, fair for 0.6 to 0.8 and bad 0.0 to 0.6. Only one bad score is observed on the table, it is below the threshold 0.6 and most of the values are close or above 0.8.

| Epitope Pair Index | HHV-6 Epitope | T1DM Epitope | HHV-6/HLA complex | T1DM/HLA complex |
| --- | --- | --- | --- | --- |
|  |  |  |  |  |
| 163076 | 0.41 | 0.69 | 0.88 | 0.87 |
| 185874 | 0.74 | 0.79 | 0.89 | 0.89 |
| 430465 | 0.73 | 0.71 | 0.85 | 0.89 |
| 431152 | 0.74 | 0.72 | 0.87 | 0.88 |
| 431175 | 0.77 | 0.77 | 0.90 | 0.87 |
| 431177 | 0.76 | 0.79 | 0.88 | 0.86 |
| 431594 | 0.74 | 0.78 | 0.88 | 0.86 |
| 448259 | 0.73 | 0.69 | 0.90 | 0.89 |
| 449222 | 0.81 | 0.80 | 0.86 | 0.86 |
| 468440 | 0.78 | 0.79 | 0.85 | 0.90 |
| 482558 | 0.63 | 0.80 | 0.85 | 0.84 |
| 488701 | 0.71 | 0.71 | - | - |
| 541490 | 0.68 | 0.81 | 0.88 | 0.85 |
| 562998 | 0.79 | 0.63 | 0.86 | 0.87 |
| 563936 | 0.72 | 0.78 | 0.84 | 0..82 |
| 571479 | 0.71 | 0.75 | 0.86 | 0.85 |
| 571480 | 0.72 | 0.78 | 0.86 | 0.87 |
| 573185 | 0.65 | 0.72 | 0.85 | 0.86 |
| 602576 | 0.77 | 0.71 | 0.91 | 0.90 |
| 620896 | 0.75 | 0.75 | 0.85 | 0.87 |
| 625331 | 0.75 | 0.74 | 0.88 | 0.88 |
| 771155 | 0.73 | 0.80 | - | - |
| 890415 | 0.77 | 0.83 | 0.89 | 0.89 |
| 935014 | 0.78 | 0.84 | 0.84 | 00.87 |
| 935131 | 0.65 | 0.76 | 0.84 | 0.86 |

**Figure S1:** Homology between epitopes in HHV-6A and HHV-6B proteins.

***Glycoprotein B***

IRAGYNHKY

IRAGYNHKY

LTDPKQTCI

LTDPKQTCI

DILYVQLQYL

DILYVQLQYL

>sp|P36319|GB_HHV6G Envelope glycoprotein B OS=Human herpesvirus 6A (strain GS) OX=10369 GN=gB PE=3 SV=1

MSKMVVLFLAVFLMNSVLMIYCDPDHYIRAGYNHKYPFRICSIAKGTDLMRFDRDISCSP

YKSNAKMSEGFFIIYKTNIETYTFPVRTYKKELTFQSSYRDVGVVYFLDRTVMGLAMPVY

EANLVNSHAQCYSAVAMKRPDGTVFSAFHEDNNKNNTLNLFPLNFKSITNKRFITTKEPY

FARGPLWLYSTSTSLNCIVTEATAKAKYPFSYFALTTGEIVEGSPFFNGSNGKHFAEPLE

KLTILENYTMIEDLMNGMNGATTLVRKIAFLEKADTLFSWEIKEENESVCMLKHWTTVTH

GLRAETDETYHFISKELTAAFVAPKESLNLTDPKQTCIKDEFEKIINEVYMSDYNDTYSM

NGSYQIFKTTGDLILIWQPLVQKSLMFLEQGSEKIRRRRDVVDVKSRHDILYVQLQYLYD

TLKDYINDALGNLAESWCLDQKRTITMLHELSKISPSSIVSEVYGRPISAQLHGDVLAIS

KCIEVNQSSVQLHKSMRVVDAKGVRSETMCYNRPLVTFSFVNSTPEVVPGQLGLDNEILL

GDHRTEECEIPSTKIFLSGNHAHVYTDYTHTNSTPIEDIEVLDAFIRLKIDPLENADFKV

LDLYSPDELSRANVFDLENILREYNSYKSALYTIEAKIATNTPSYVNGINSFLQGLGAIG

TGLGSVISVTAGALGDIVGGVVSFLKNPFGGGLMLILAIVVVVIIIVVFVRQRHVLSKPI

DMMFPYATNPVTTVSSVTGTTVVKTPSVKDVDGGTSVAVSEKEEGMADVSGQVSDDEYSQ

EAALKMLKAIKSLDESYRRKPSSSESHASKPSLIDRIRYRGYKSVNVEEA

>sp|P36320|GB_HHV6Z Envelope glycoprotein B OS=Human herpesvirus 6B (strain Z29) OX=36351 GN=gB PE=3 SV=2

MSKMRVLFLAVFLMNSVLMIYCDSDDYIRAGYNHKYPFRICSIAKGTDLMRFDRDISCSP

YKSNAKMSEGFFIIYKTNIETYTFPVRTYKNELTFPTSYRDVGVVYFLDRTVMGLAMPVY

EANLVNSRAQCYSAVAIKRPDGTVFSAYHEDNNKNETLELFPLNFKSVTNKRFITTKEPY

FARGPLWLYSTSTSLNCIVTEATAKAKYPFSYFALTTGEIVEGSPFFDGSNGKHFAEPLE

KLTILENYTMIEDLMNGMNGATTLVRKIAFLEKGDTLFSWEIKEENESVCMLKHWTTVTH

GLRAETDETYHFISKELTAAFVASKESLNLTDPKQTCIKNEFEKIITDVYMSDYNDAYSM

NGSYQIFKTTGDLILIWQPLVQKSLMVLEQGSVNLRRRRDLVDVKSRHDILYVQLQYLYD

TLKDYINDALGNLAESWCLDQKRTITMLHELSKISPSSIVSEVYGRPISAQLHGDVLAIS

KCIEVNQSSVQLYKSMRVVDAKGVRSETMCYNRPLVTFSFVNSTPEVVLGQLGLDNEILL

GDHRTEECEIPSTKIFLSGNHAHVYTDYTHTNSTPIEDIEVLDAFIRLKIDPLENADFKL

LDLYSPDELSRANVFDLENILREYNSYKSALYTIEAKIATNTPSYVNGINSFLQGLGAIG

TGLGSVISVTAGALGDIVGGVVSFLKNPFGGGLMLILAIVVVVIIIVVFVRQKHVLSKPI

DMMFPYATNPVTTVSSVTGTTVVKTPSVKDADGGTSVAVSEKEEGMADVSGQISGDEYSQ

EDALKMLKAIKSLDESYRRKPSSSESHASKPSLIDRIRYRGYKSVNVEEA

**Glycoprotein H**

SSHCRNGNF

**E**SHC**K**NGN**S**

VVEVRQMQY

VVEVRQMQY

TSINFKAPY

T**D**INFKAPY

>sp|P68323|GH_HHV6G Envelope glycoprotein H OS=Human herpesvirus 6A (strain GS) OX=10369 GN=gH PE=3 SV=1

MLLRLWVFVLLTPCYGWRPLNISNSSHCRNGNFENPIVRPGFITFNFYTKNDTRIYQVPK

CLLGSDITYHLFDAINTTESLTNYEKRVTRFYEPPMNDILRLSPVPSVKQFNLDRSIQPQ

VVYSLNMYPSQGIYYVRVVEVRQMQYDNVSCKLPNSLKELIFPVQVRCAKITRYVGEDIY

THFFTPDFMILYIQNPAGDLTMMYGNTTSINFKAPYKKSSFIFKQTLTDDLLLIVEKDVI

DVQYRFISDATFVDETLNDVDEVEALLLKFNNLGIQTLLRGDCKKPNYAGIPQMMFLYGI

VHFSYSTKNTGPMPVLRVLKTHENLLSIDSFVNRCVNVSEGTLQYPKMKEFLKYEPSDYS

YITKNKSISVSTLLTYLATAYESNVTISKYKWTDIANTLQNIYEKHMFFTNLTFSDRETL

FMLAEIANIIPTDERMQRHMQLLIGNLCNPVEIVSWARMLTADRAPNLENIYSPCASPVR

RDVTNSFLKTVLTYASLDRYRSDMMEMLSVYRPPNMERVAAIQCLSPSEPAASLTLPNVT

FVISPSYVIKGVSLTITTTIVATSIIITAIPLNSTCVSTNYKYAGQDLLVLRNISSQTCE

FCQSVVMEYDDIDGPLQYIYIKNIDELKTLTDPNNNLLVPNTRTHYLLLAKNGSVFEMSE

VGIDIDQVSIILVIIYILIAIIALFGLYRLIRLC

>sp|P52543|GH_HHV6Z Envelope glycoprotein H OS=Human herpesvirus 6B (strain Z29) OX=36351 GN=gH PE=3 SV=1

MLFRLWVFVLLTPCYSWRPWTISDESHCKNGNSENPIVRPGFITFNFYTKNDTRIYQVPK

CLLGSDITYHLFDAINTTESLTNYEKRVTRFYEPPMNDILRLSTVPAVKQFNLDHSIQPQ

IVYSLNLYPSHGIYYIRVVEVRQMQYDNVSCKLPNSLNELIFPVQVRCAKITRYAGENIY

THFFTPDFMILYIQNPAGDLTMMYGNTTDINFKAPYRKSSFIFKQTLTDDLLLIVEKDVV

DEEYRFISDATFVDETLDDVDEVEALLLKFNNLGIQTLLRGDCKKPDYAGIPQMMFLYGI

VHFSYSTKNTGPMPVLRVLKTHENLLSIDSFVNRCVNVSEGTIQYPKMKEFLKYEPSDYS

YITKNKSIPVSTLLTYLATAYETNVTISRYKWSDIANTLQKIYEKHMFFTNLTFSDRETL

FMLAEIANFIPADERMQRHMQLLIGNLCNPVEIVSWAHMLTADKAPNLENIYSPCASPVR

RDVTNSFVKTVLTYASLDRYRSDMMEMLSVYRPPDMARVAAIQCLSPSEPAASLPLPNVT

FVISPSYVIKGVSLTITTTIVATSIIITAIPLNSTCVSTNYKYAGQDLLVLRNISSQTCE

FCQSVVMEYDDIDGPLQYIYIKNIDELKTLTDPNNNLLVPNTRTHYLLLAKNGSVFEMSE

VGIDIDQVSIILVIIYVLIAIIALFGLYRLIRLC

**Glycoprotein Q**

LTAMTAIAF

LTAMTAIAF

NLTTTNIKY

**K**LTTTNIKY

NIFTVQGRY

NIFTVQARY

>sp|Q69572|GQ1_HHV6U Glycoprotein Q1 OS=Human herpesvirus 6A (strain Uganda-1102) OX=10370 GN=U100 PE=1 SV=4

MATARLSAMKPPRSCALIFLCAFSMATAPTNATAHRRAGTVKSTPPPEDKHSYTAKYYDK

DIYFNIYEGRNSTPRRRTLSEIISKFSTSEMLSLKRVKAFVPVDENPTTTLEDIADILNY

AVCDDNSCGCTIETQARIMFGDIIICVPLSADNKGVRNFKDRIMPKGLSQILSSSLGLHL

SLLYGAFGSNYNSLAYMRRLKPLTAMTAIAFCPMTTKLELRQNYKVKETLCELIVSIEIL

KIRNNGGQTMKTLTSFAIVRKDNDGQDWETCTRFAPVNIEDILRYKRVANDTCCRHRDVQ

HGRRTLESSNSWTQTQYFEPWQDIVDVYVPINDTHCPNDSYVVFETLQGFEWCSRLNKNE

TKNYLSSVLGFRNALFETEELMETIAMRLASQILSMVGQQGTTIRDIDPAIVSALWHSLP

ENLTTTNIKYDIASPTHMAPALCTIFVQTGTSKQRFRNAGLLMVNNIFTVQGRYTTQNMF

ERKEYVYKHLGQALCQDGEILFQNEGQKFCRPLTDNRTIVYTMQDQVQKPLSVTWMDFNL

VISDYGRDVINNLTKSAMLARKNGPRYLQMENGPRYLQMETFISDLFRHECYQDNYYVLD

KKLQMFYPTTHSNELLFYPSEATLPSPWQEPPFSSPWPEPTFPSRWYWLLLNYTNY

>sp|Q9QJ11|GQ1_HHV6Z Glycoprotein Q1 OS=Human herpesvirus 6B (strain Z29) OX=36351 GN=U100 PE=1 SV=3

MRPPRRSAPILVCAISMATALSNATVHRDAGTVESTPPPDDEDNYTAKYYDDSIYFNIYD

GTNPTPRRRTLPEIISKFSTSEMSRLGGLKAFVPVDYTPTTTLEDIEDLLNYAICDDNSC

GCLIETEARIMFGDIIICVPLSAESRGVRNLKSRIMPMGLSQILSSGLGLHFSLLYGAFG

SNYNSLAYMERLKPLTAMTAIAFCPMTSKLELRQNYRLEKARCELIVNIELLKIQNHGGQ

TIKTLTSFAIVRKDSDGQDWETCTRFASVSIEDILRSKPAANGTCCPPRDVHHDRPTLQS

SNSWTRTEYFEPWQDVVDAYVPINDNHCPNDSYVVFQTLQGHEWCSRLNKNDTKNYLSSV

LAFKNALYETEELMETIGMRLASQILSLVGQRGTSIRNIDPAIVSALWHSLPEKLTTTNI

KYDIASPTHMSPALCTIFIQTGTSKQRFRNAGLLMVNNIFTVQARYSKQNMFEKKIYGYE

HLGQALCEDGEILFQNAGQKFCRPFTDNRTIVYTMQDQVQRPWSVTWMDFNLVISDYGRA

VIENLTESAMSAHKNGPRYLQMETFISDLFRYECHRDNRYVLEKKLQMFYPTTHMNELLF

YPSDPTLPSPYGNGHY

**Figure S2:** Details of the Boltz-2 structure of HLA-A-, HLA-B- nd HLA-DRB5-01-01 structures.

**HLA-A:01:01**

MAVMAPRTLLLLLSGALALTQTWAGSHSMRYFFTSVSRPGRGEPRFIAVGYVDDTQFVRFDSDAASQKMEPRAPWIEQEGPEYWDQETRNMKAHSQTDRANLGTLRGYYNQSEDGSHTIQIMYGCDVGPDGRFLRGYRQDAYDGKDYIALNEDLRSWTAADMAAQITKRKWEAVHAAEQRRVYLEGRCVDGLRRYLENGKETLQRTDPPKTHMTHHPISDHEATLRCWALGFYPAEITLTWQRDGEDQTQDTELVETRPAGDGTFQKWAAVVVPSGEEQRYTCHVQHEGLPKPLTLRWELSSQPTIPIVGIIAGLVLLGAVITGAVVAAVMWRRKSSDRKGGSYTQAASSDSAQGSDVSLTACKV


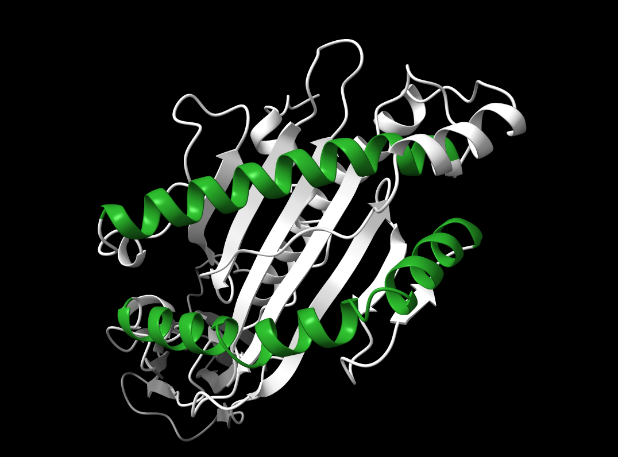

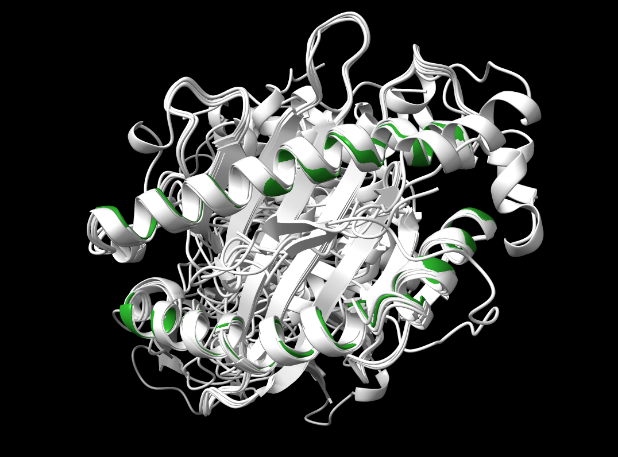


Matchmaker HLA-A-01-01_model_0.cif, chain B (#1) with HLA-A-08-01_model_0.cif, chain B (#2), sequence alignment score = 1573.2

RMSD between 174 pruned atom pairs is 0.614 angstroms; (across all 362 pairs: 13.766)

Matchmaker HLA-A-01-01_model_0.cif, chain B (#1) with HLA-A-23-01_model_0.cif, chain B (#3), sequence alignment score = 1754.2

RMSD between 183 pruned atom pairs is 0.573 angstroms; (across all 365 pairs: 12.292)

Matchmaker HLA-A-01-01_model_0.cif, chain B (#1) with HLA-A-25-01_model_0.cif, chain B (#4), sequence alignment score = 1710.9

RMSD between 180 pruned atom pairs is 0.581 angstroms; (across all 365 pairs: 14.986)

Matchmaker HLA-A-01-01_model_0.cif, chain B (#1) with HLA-A-26-01_model_0.cif, chain B (#5), sequence alignment score = 1710.2

RMSD between 179 pruned atom pairs is 0.486 angstroms; (across all 365 pairs: 15.259)

**HLA-B-08-01**

MLVMAPRTVLLLLSAALALTETWAGSHSMRYFDTAMSRPGRGEPRFISVGYVDDTQFVRFDSDAASPREEPRAPWIEQEGPEYWDRNTQIFKTNTQTDRESLRNLRGYYNQSEAGSHTLQSMYGCDVGPDGRLLRGHNQYAYDGKDYIALNEDLRSWTAADTAAQITQRKWEAARVAEQDRAYLEGTCVEWLRRYLENGKDTLERADPPKTHVTHHPISDHEATLRCWALGFYPAEITLTWQRDGEDQTQDTELVETRPAGDRTFQKWAAVVVPSGEEQRYTCHVQHEGLPKPLTLRWEPSSQSTVPIVGIVAGLAVLAVVVIGAVVAAVMCRRKSSGGKGGSYSQAACSDSAQGSDVSLTA


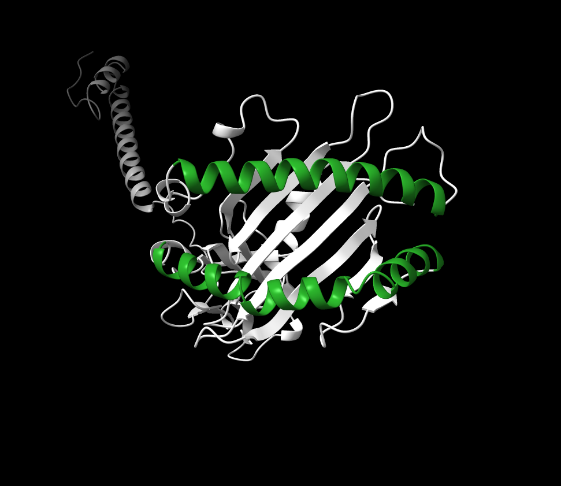

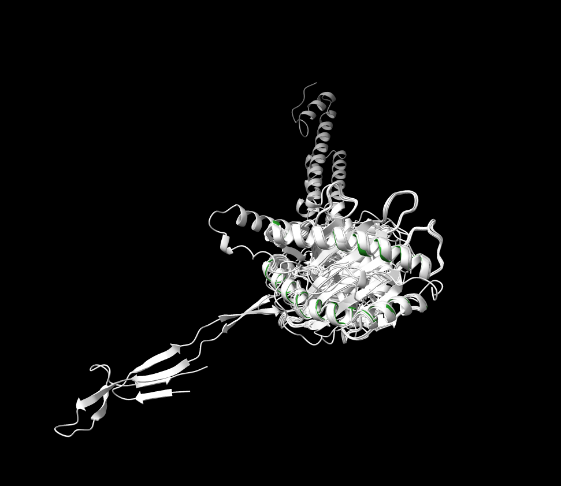


Matchmaker HLA-B-08-01_model_0.cif, chain B (#1) with HLA-B-15-10_model_0.cif, chain B (#2), sequence alignment score = 1667.2

RMSD between 172 pruned atom pairs is 0.757 angstroms; (across all 362 pairs: 37.711)

Matchmaker HLA-B-08-01_model_0.cif, chain B (#1) with HLA-B-18-01_model_0.cif, chain B (#3), sequence alignment score = 1755.1

RMSD between 186 pruned atom pairs is 0.906 angstroms; (across all 362 pairs: 34.183)

Matchmaker HLA-B-08-01_model_0.cif, chain B (#1) with HLA-B-38-01_model_0.cif, chain B (#4), sequence alignment score = 1752.7

RMSD between 274 pruned atom pairs is 0.890 angstroms; (across all 362 pairs: 36.264)

Matchmaker HLA-B-08-01_model_0.cif, chain B (#1) with HLA-B-38-02_model_0.cif, chain B (#5), sequence alignment score = 1709.2

RMSD between 202 pruned atom pairs is 0.921 angstroms; (across all 362 pairs: 36.279)

Matchmaker HLA-B-08-01_model_0.cif, chain B (#1) with HLA-B-44-02_model_0.cif, chain B (#6), sequence alignment score = 1740.5

RMSD between 219 pruned atom pairs is 0.804 angstroms; (across all 362 pairs: 9.263)

Matchmaker HLA-B-08-01_model_0.cif, chain B (#1) with HLA-B-57-01_model_0.cif, chain B (#7), sequence alignment score = 1503.2

RMSD between 172 pruned atom pairs is 0.501 angstroms; (across all 362 pairs: 46.740)

**HLA-DRB5-01-01**

MVCLKLPGGSYMAKLTVTLMVLSSPLALAGDTRPRFLQQDKYECHFFNGTERVRFLHRDIYNQEEDLRFDSDVGEYRAVTELGRPDAEYWNSQKDFLEDRRAAVDTYCRHNYGVGESFTVQRRVEPKVTVYPARTQTLQHHNLLVCSVNGFYPGSIEVRWFRNSQEEKAGVVSTGLIQNGDWTFQTLVMLETVPRSGEVYTCQVEHPSVTSPLTVEWRAQSESAQSKMLSGVGGFVLGLLFLGAGLFIYFKNQKGHSGLHPTGLVS


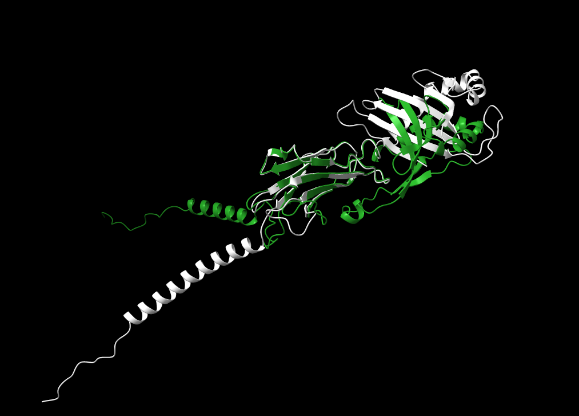

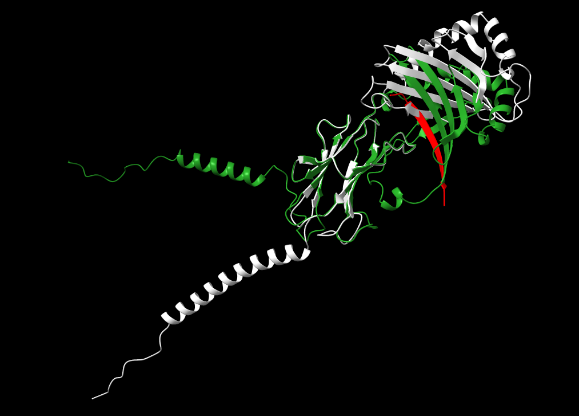


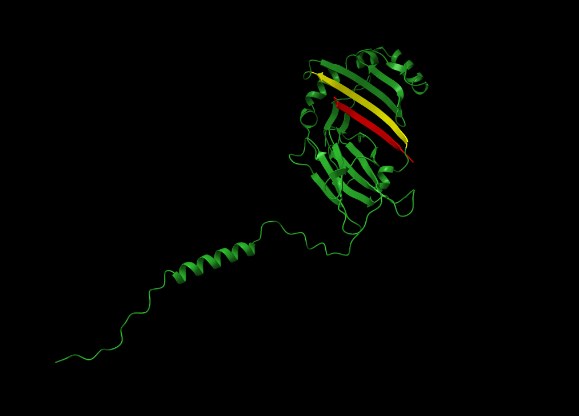


HLA-DRB5-01-01 is grey

HLA-DRB5-01-01 plus HHV-6B epitope is green

HHV-6B epitope is red

Matchmaker HLA-DRB5-01-01_model_0.cif, chain A (#1) with epitope_7_herpesvirus6_model_0.cif, chain A (#2), sequence alignment score = 1286.5

RMSD between 95 pruned atom pairs is 0.538 angstroms; (across all 266 pairs: 22.295)

**Figure S3:** Epitope Pairs Conformations and biding to HLAs.

Epitope Pair 163076: Conformation of the pair epitopes, free (L) and bound (R) and corresponding Boltz-2 Confidence Scores for HHV-6 (red), T1DM (green), HHV-6/ HLA-A*25:01 (white), T1DM/ HLA-A*25:01 (white): 0.41, 0.69, 0.88, 0.87.


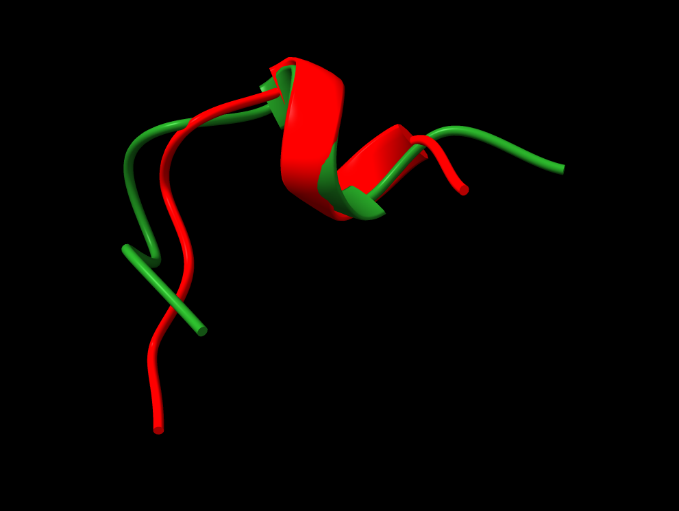

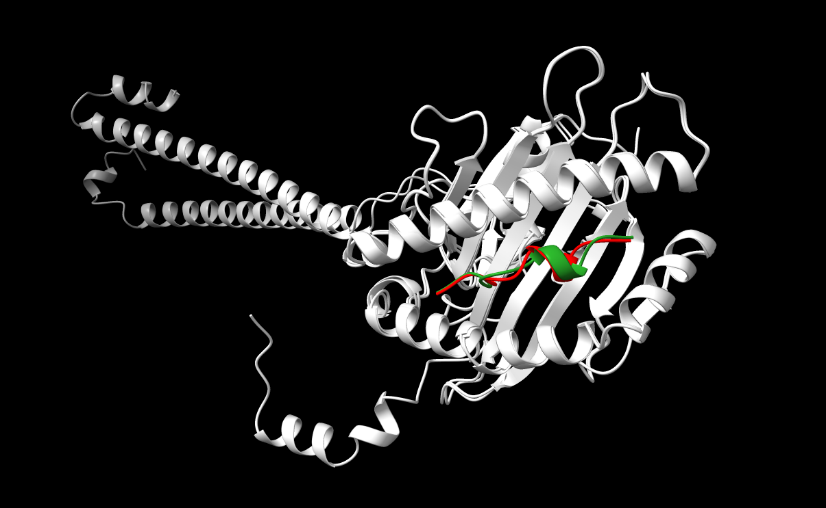


Epitope Pair 185874: Conformation of the pair epitopes, free (L) and bound (R) and corresponding Boltz-2 Confidence Scores for HHV-6 (red), T1DM (green), HHV-6/ HLA-A*25:01 (white), T1DM/ HLA-A*25:01 (white): 0.74, 0.79, 0.89, 0.89.

**
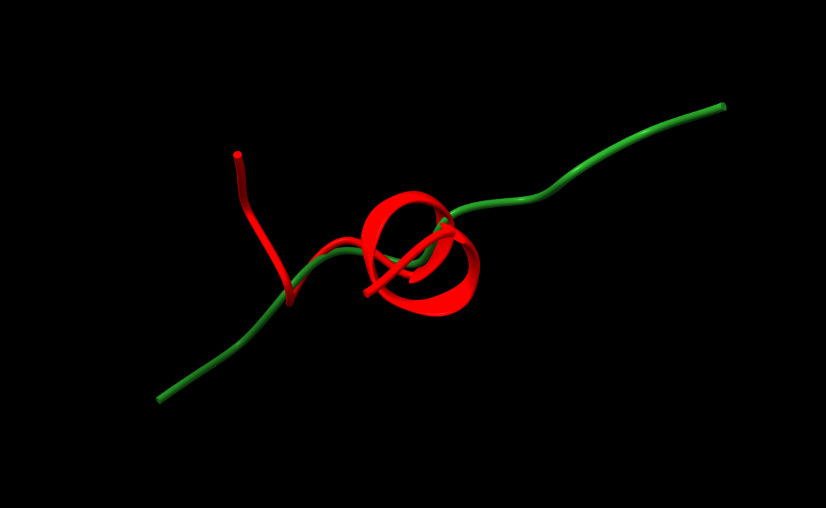

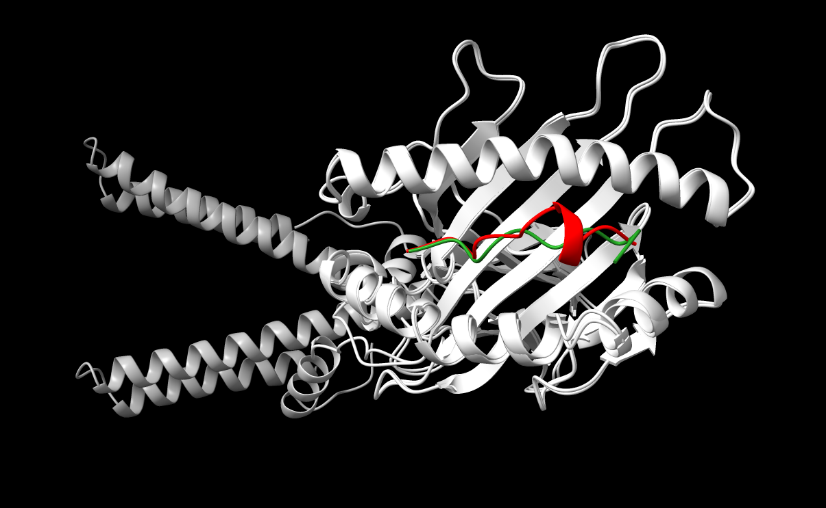
**

Epitope Pair 430465: Conformation of the pair epitopes, free (L) and bound (R) and corresponding Boltz-2 Confidence Scores for HHV-6 (red), T1DM (green), HHV-6/ HLA-A*25:01 (white), T1DM/ HLA-A*25:01 (white): 0.73, 0.71, 0.85, 0.87.

**
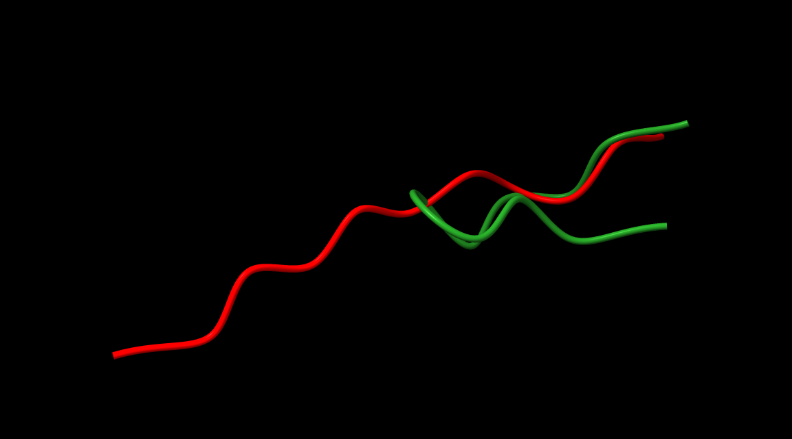

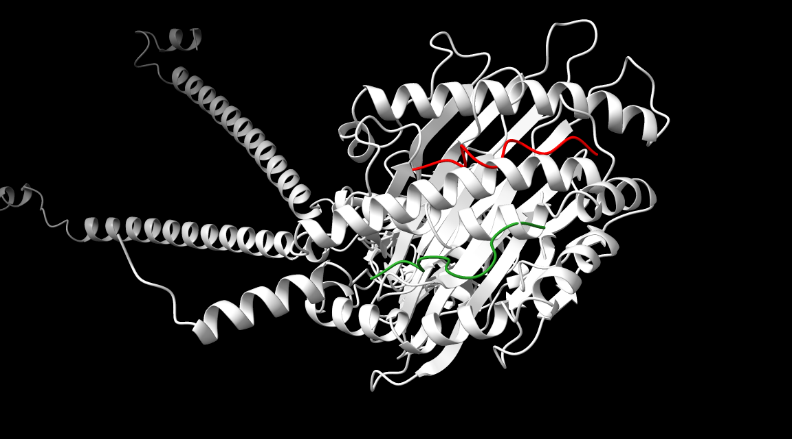
**

Epitope Pair 431152: Conformation of the pair epitopes, free (L) and bound (R) and corresponding Boltz-2 Confidence Scores for HHV-6 (red), T1DM (green), HHV-6/ HLA-A*25:01 (white), T1DM/ HLA-A*25:01 (white): 0.74, 0.72, 0.87, 0.88.

**
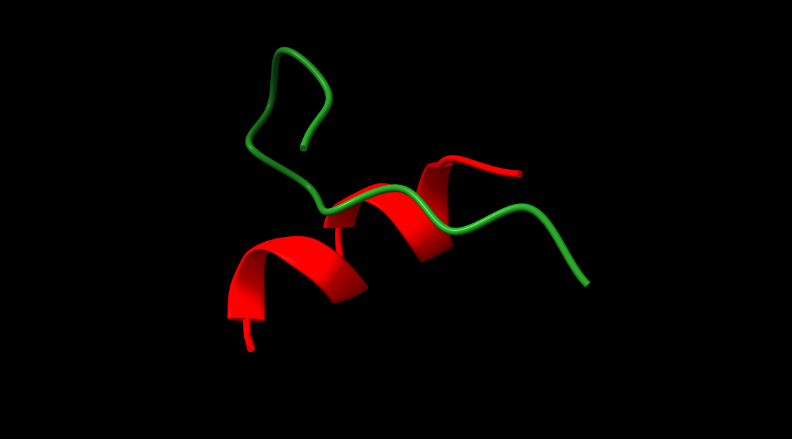

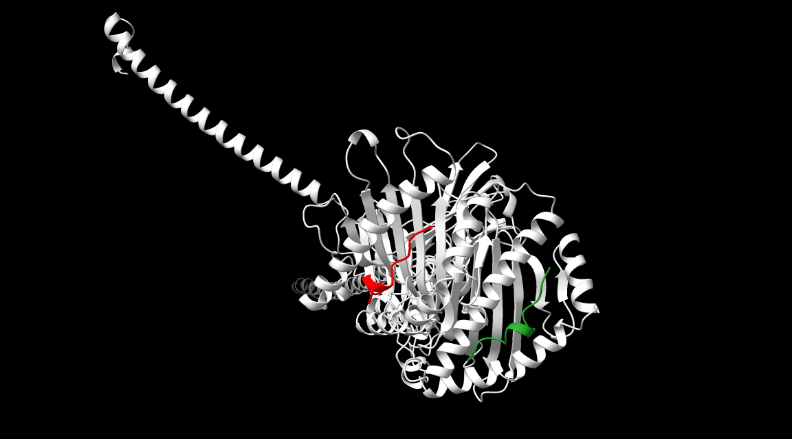
**

Epitope Pair 431175: Conformation of the pair epitopes, free (L) and bound (R) and corresponding Boltz-2 Confidence Scores for HHV-6 (red), T1DM (green), HHV-6/ HLA-A*01:01 (white), T1DM/ HLA-A*01:01 (white): 0.77, 0.77, 0.90, 0.87.

**
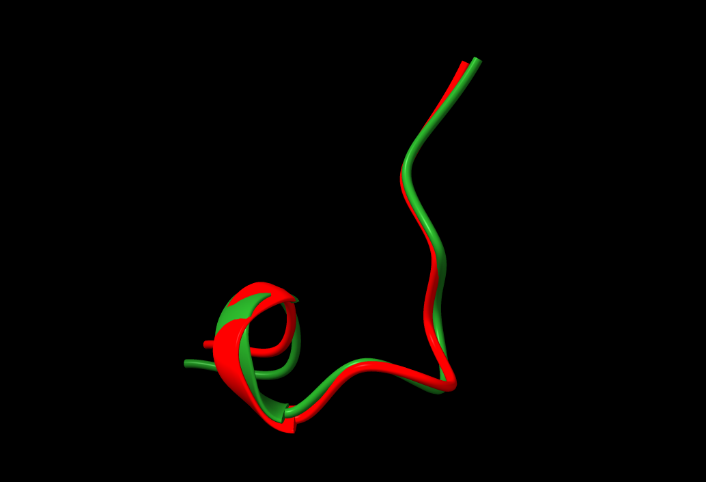

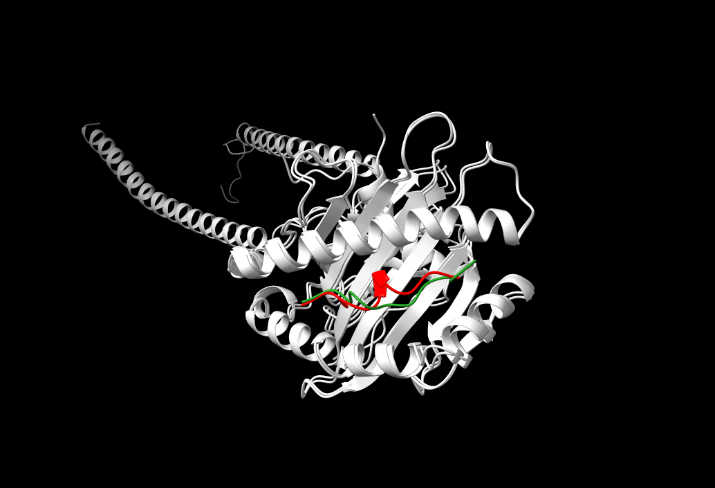
**

Epitope Pair 431177**:** Conformation of the pair epitopes, free (L) and bound (R) and corresponding Boltz-2 Confidence Scores for HHV-6 (red), T1DM (green), HHV-6/ HLA-A*25:01 (white), T1DM/ HLA-A*25:01 (white): 0.76, 0.79, 0.88, 0.86.

**
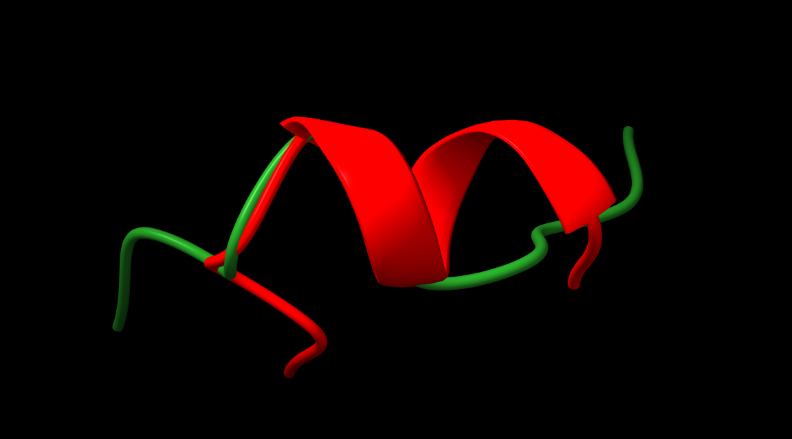

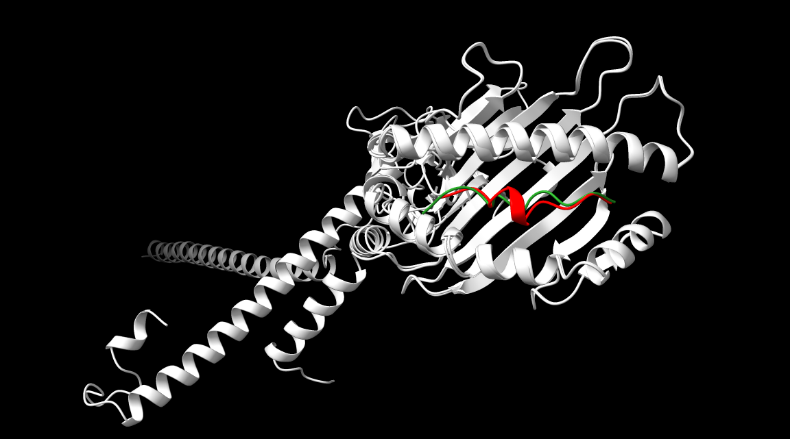
**

Epitope Pair 431594: Conformation of the pair epitopes, free (L) and bound (R) and coresponding Boltz-2 Confidence Scores for HHV-6 (red), T1DM (green), HHV-6/ HLA-A*01:01 (white), T1DM/ HLA-A*01:01 (white): 0.74, 0.78, 0.88, 0.86.

**
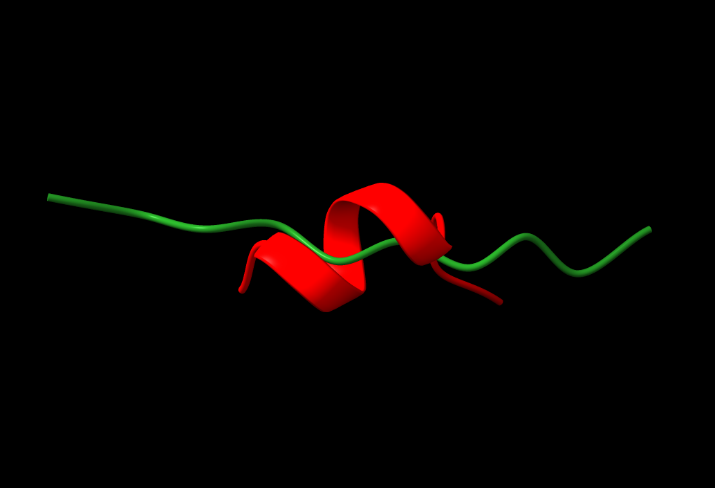

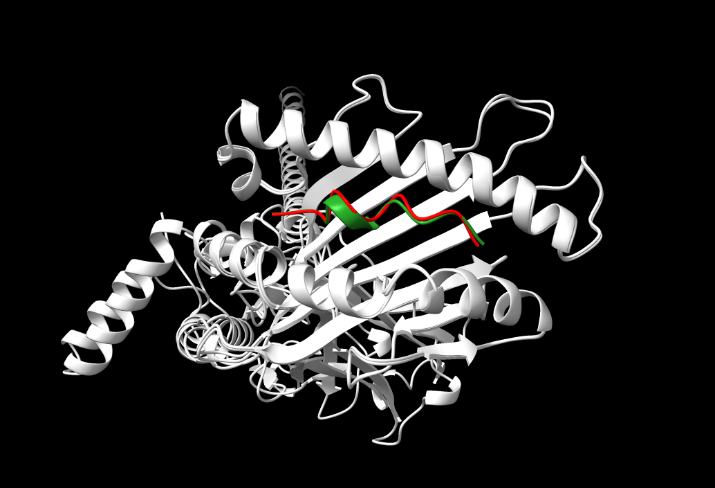
**

Epitope Pair 448259: Conformation of the pair epitopes, free (L) and bound (R) and coresponding Boltz-2 Confidence Scores for HHV-6 (red), T1DM (green), HHV-6/ HLA-A*25:01 (white), T1DM/ HLA-A*25:01 (white): 0.73, 0.69, 0.90, 0.89.

**
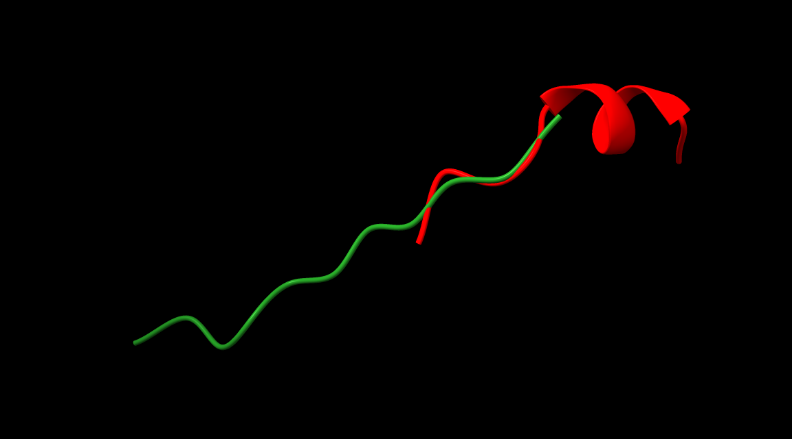

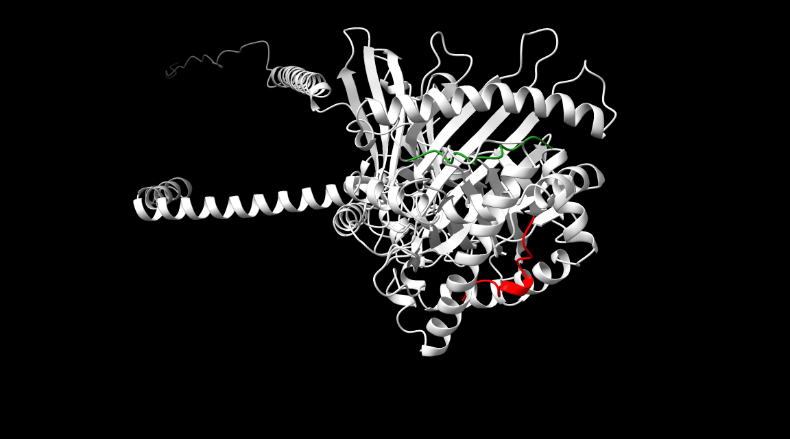
**

Epitope Pair 449222: Conformation of the pair epitopes, free (L) and bound (R) and corresponding Boltz-2 Confidence Scores for HHV-6 (red), T1DM (green), HHV-6/ HLA-A*25:01 (white), T1DM/ HLA-A*25:01 (white): 0.81, 0.80, 0.86, 0.86.

**
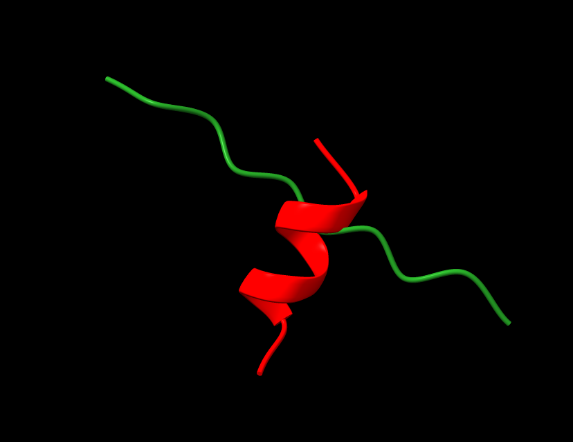

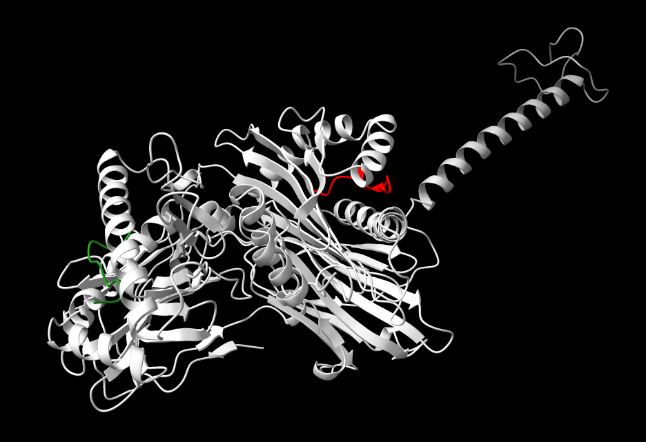
**

Epitope Pair 468440: Conformation of the pair epitopes, free (L) and bound (R) and corresponding Boltz-2 Confidence Scores for HHV-6 (red), T1DM (green), HHV-6/ HLA-A*01:01 (white), T1DM/ HLA-A*01:01 (white): 0.78, 0.79, 0.85, 0.90.

**
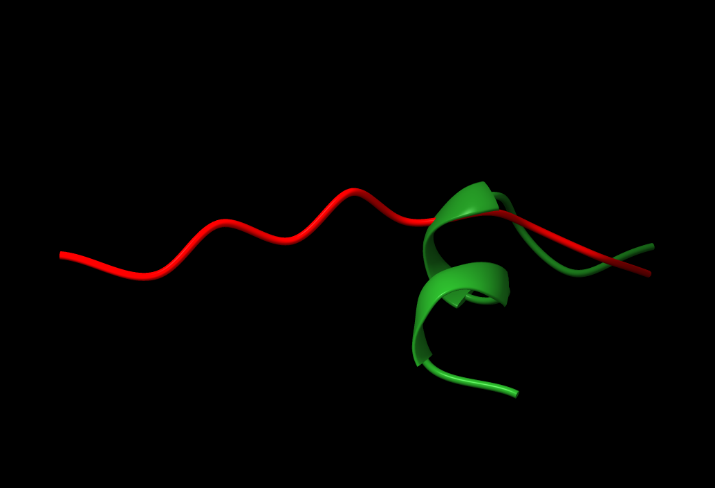

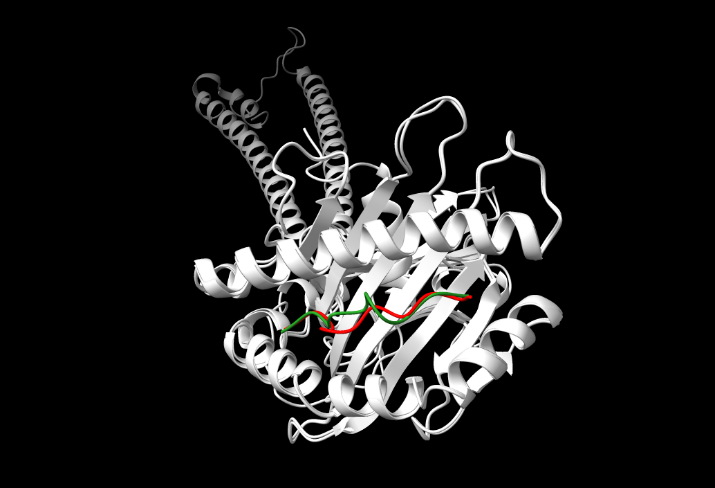
**

Epitope Pair 482558: Conformation of the pair epitopes, free (L) and bound (R) and corresponding Boltz-2 Confidence Scores for HHV-6 (red), T1DM (green), HHV-6/ HLA-B*18:01 (white), T1DM/ HLA-B*18:01 (white): 0.63, 0.80, 0.85, 0.84.

**
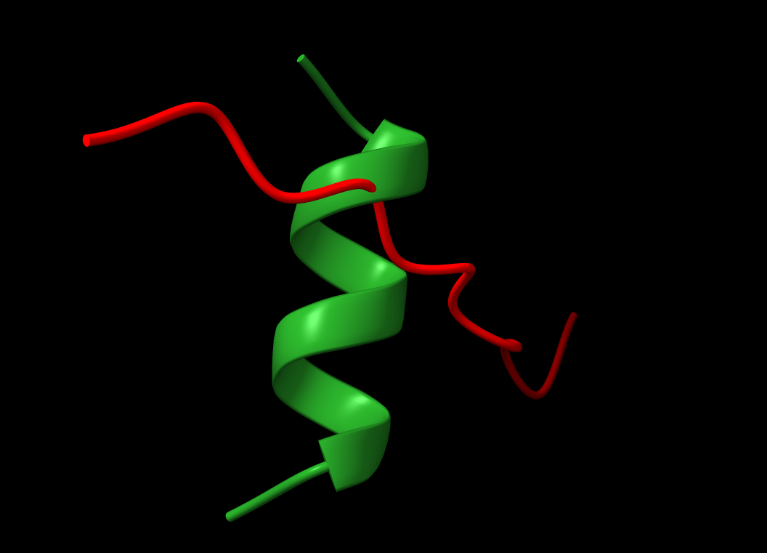

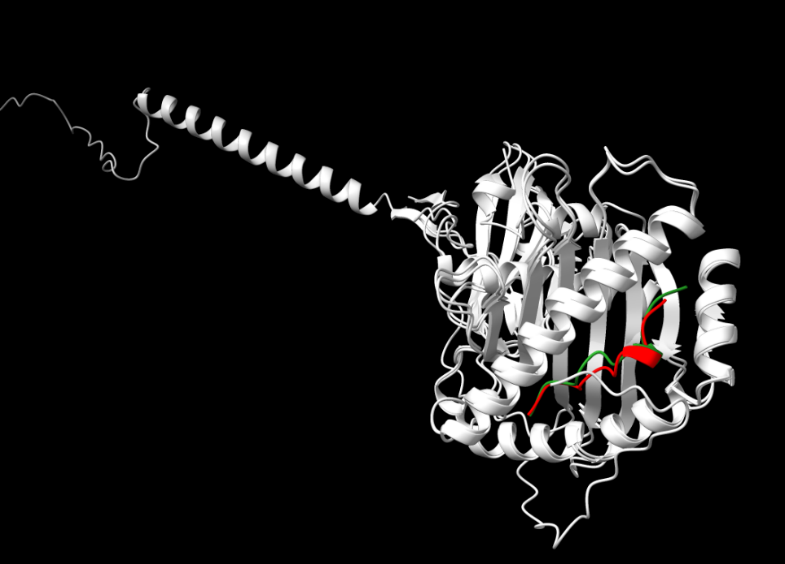
**

Epitope Pair 488701: Conformation of the pair epitopes, free and corresponding Boltz-2 Confidence Scores for HHV-6 (red), T1DM (green): 0.71, 0.77.


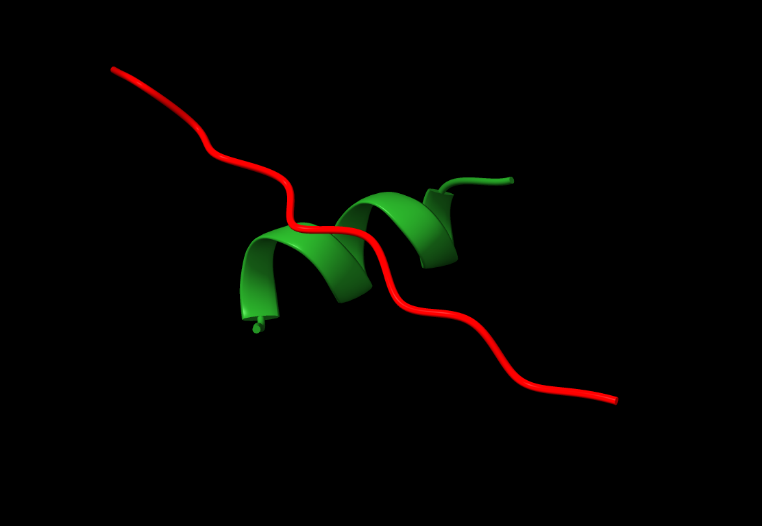


Epitope Pair 541490: Conformation of the pair epitopes, free (L) and bound (R) and corresponding Boltz-2 Confidence Scores for HHV-6 (red), T1DM (green), HHV-6/ HLA-B*08:01 (white), T1DM/ HLA-B*08:01 (white): 0.68, 0.81, 0.88, 0.85.

**
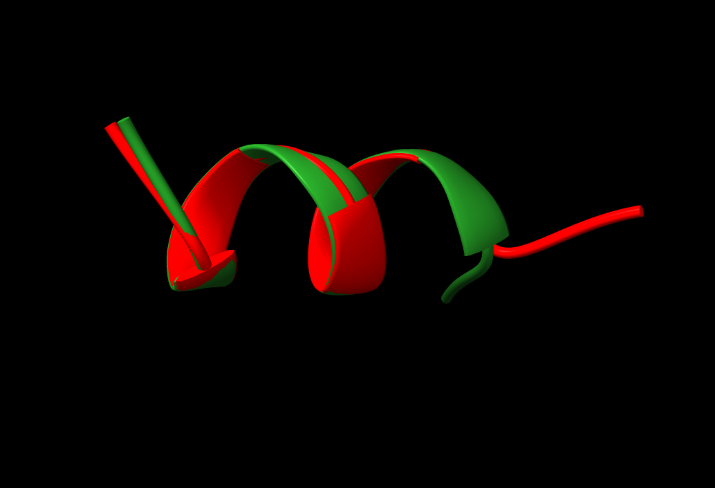

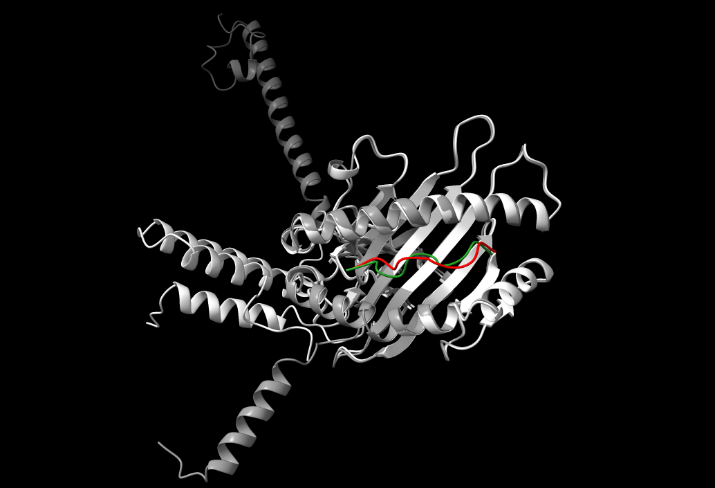
**

Epitope Pair 562998: Conformation of the pair epitopes, free (L) and bound (R) and corresponding Boltz-2 Confidence Scores for HHV-6 (red), T1DM (green), HHV-6/ HLA-A*25:01 (white), T1DM/ HLA-A*25:01 (white): 0.79, 0.63, 0.86, 0.87.

**
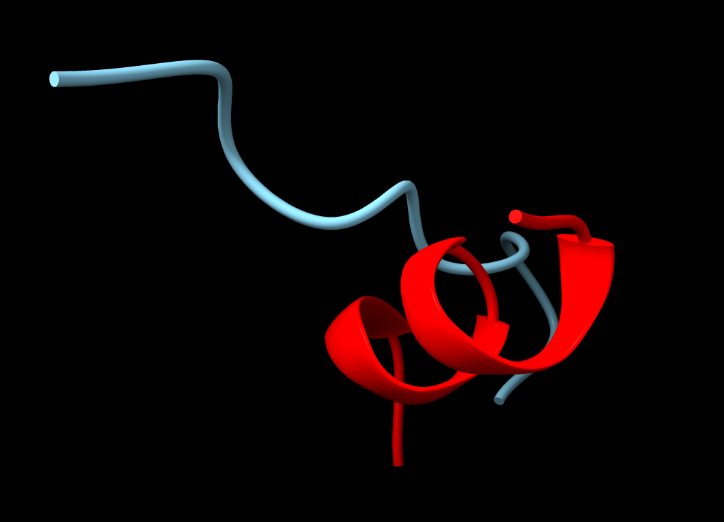

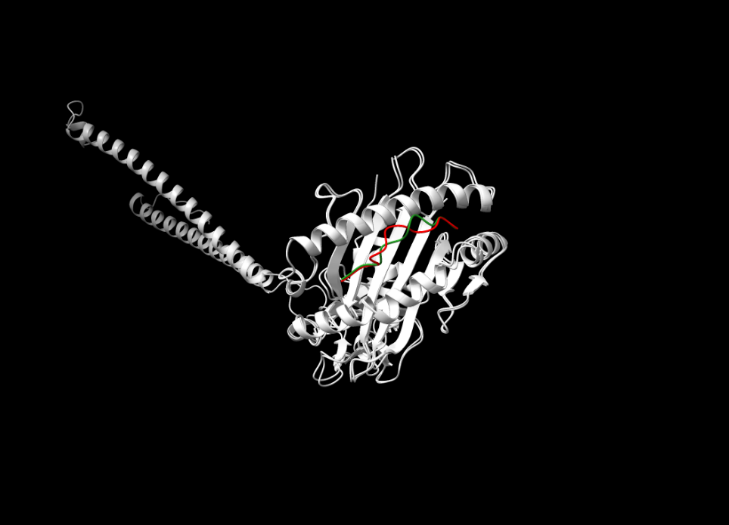
**

Epitope Pair 563936: Conformation of the pair epitopes, free (L) and bound (R) and corresponding Boltz-2 Confidence Scores for HHV-6 (red), T1DM (green), HHV-6/ HLA-A*25:01 (white), T1DM/ HLA-A*25:01 (white): 0.72, 0.78, 0.84, 0.82.

**
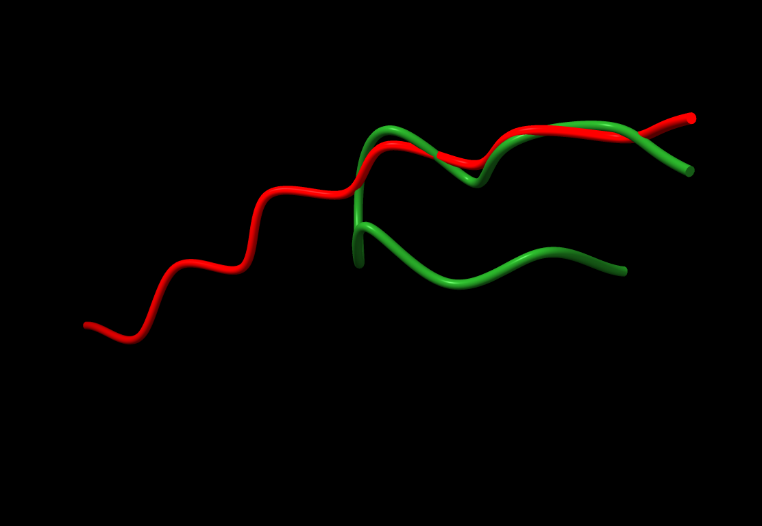

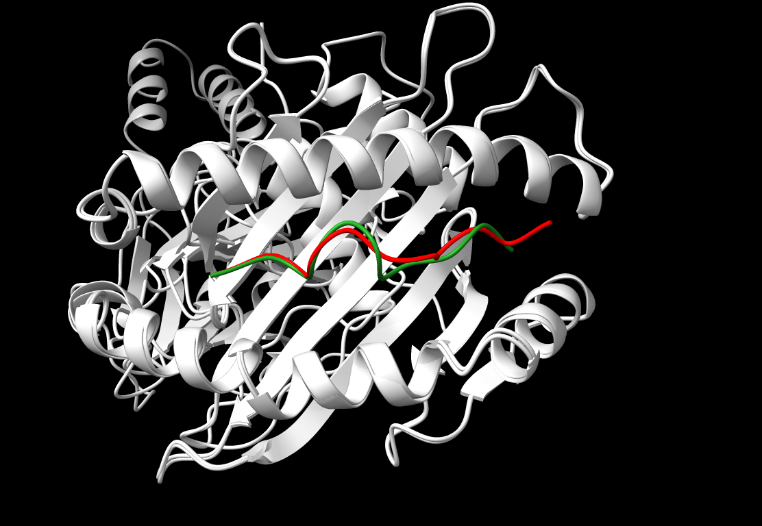
**

Epitope Pair 571479: Conformation of the pair epitopes, free (L) and bound (R) and corresponding Boltz-2 Confidence Scores for HHV-6 (red), T1DM (green), HHV-6/ HLA-A*08:01 (white), T1DM/ HLA-A*08:01 (white): 0.71, 0.75, 0.86, 0.85.

**
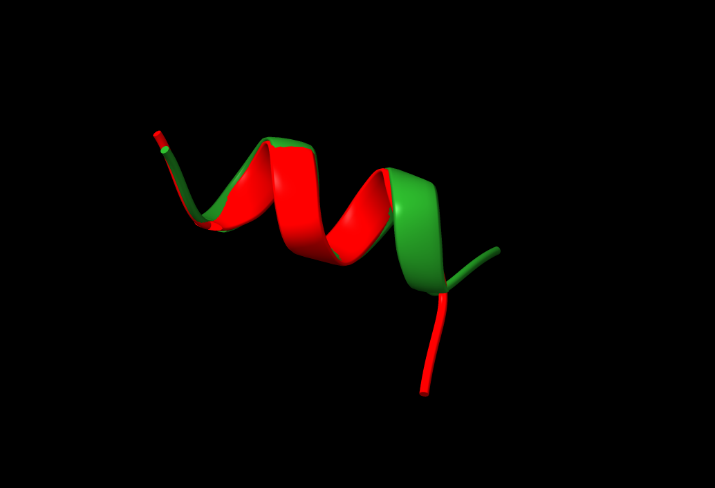

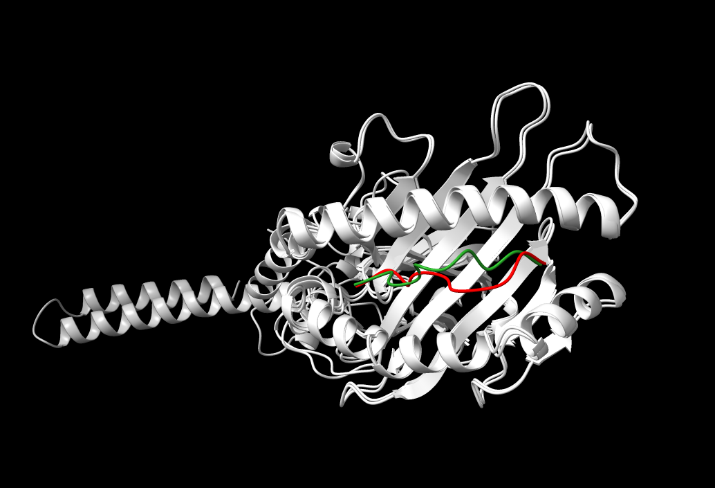
**

Epitope Pair 571480: Conformation of the pair epitopes, free (L) and bound (R) and corresponding Boltz-2 Confidence Scores for HHV-6 (red), T1DM (green), HHV-6/ HLA-A*08:01 (white), T1DM/ HLA-A*08:01 (white): 0.72, 0.78, 0.86, 0.87.

**
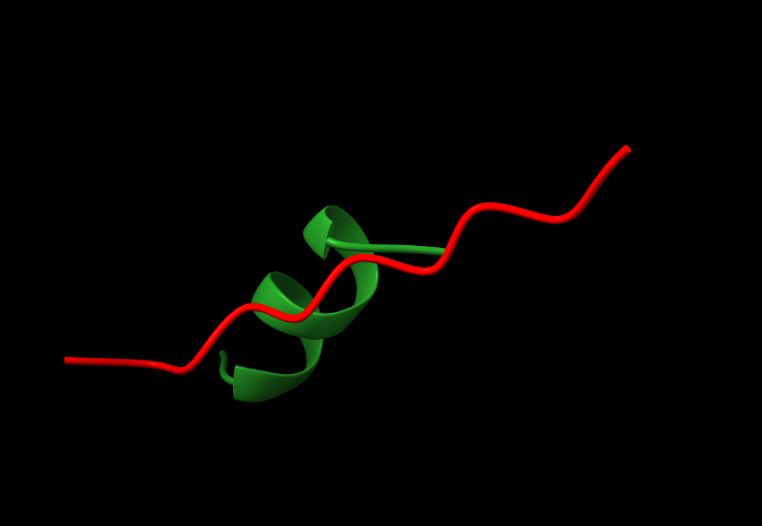

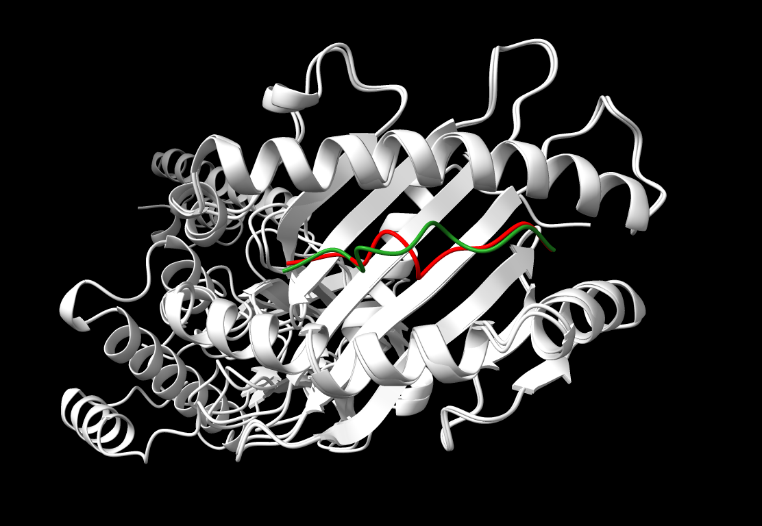
**

Epitope Pair 573185: Conformation of the pair epitopes, free (L) and bound (R) and corresponding Boltz-2 Confidence Scores for HHV-6 (red), T1DM (green), HHV-6/ HLA-A*08:01 (white), T1DM/ HLA-A*08:01 (white): 0.65, 0.72, 0.85, 0.86.

**
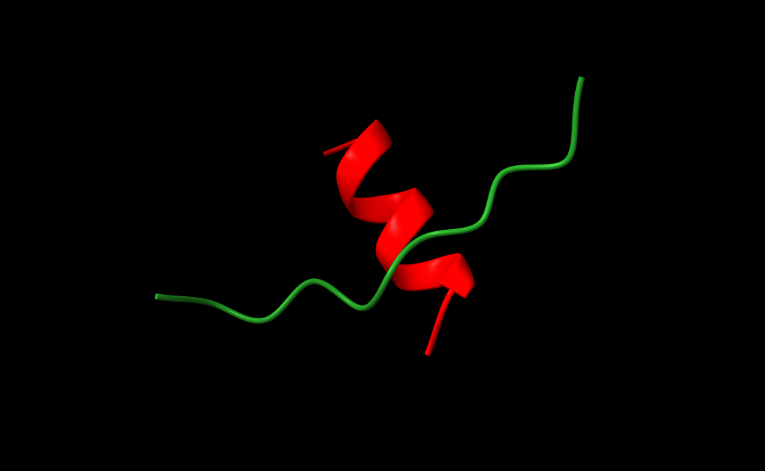

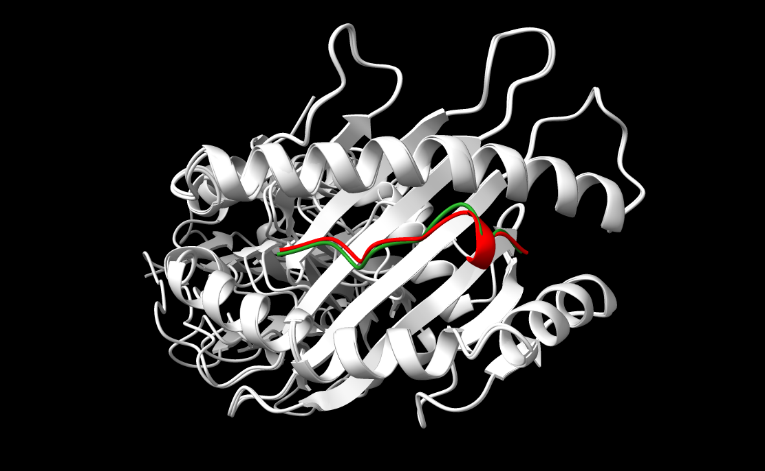
**

Epitope Pair 602576: Conformation of the pair epitopes, free (L) and bound (R) and corresponding Boltz-2 Confidence Scores for HHV-6 (red), T1DM (green), HHV-6/ HLA-A*08:01 (white), T1DM/ HLA-A*08:01 (white): 0.77, 0.71, 0.91, 0.90.

**
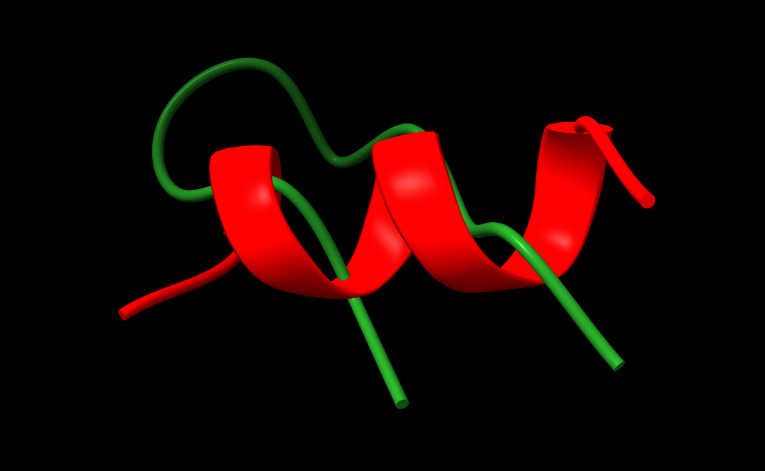

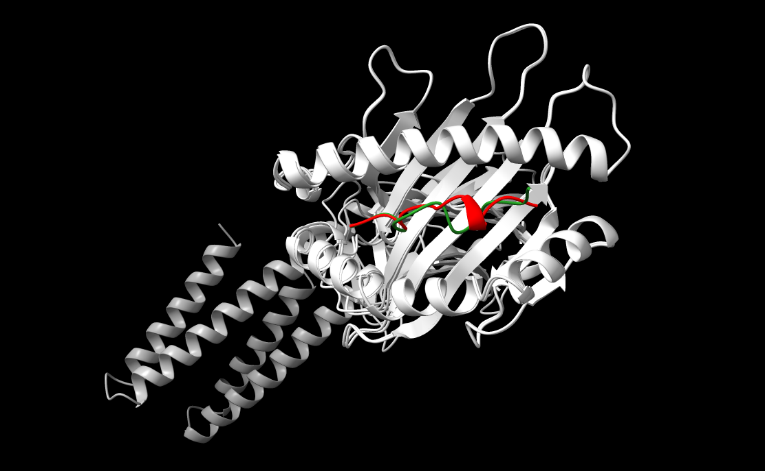
**

Epitope Pair 620896**:** Conformation of the pair epitopes, free (L) and bound (R) and corresponding Boltz-2 Confidence Scores for HHV-6 (red), T1DM (green), HHV-6/ HLA-A*23:01 (white), T1DM/ HLA-A*23:01 (white): 0.75, 0.75, 0.85, 0.87.

**
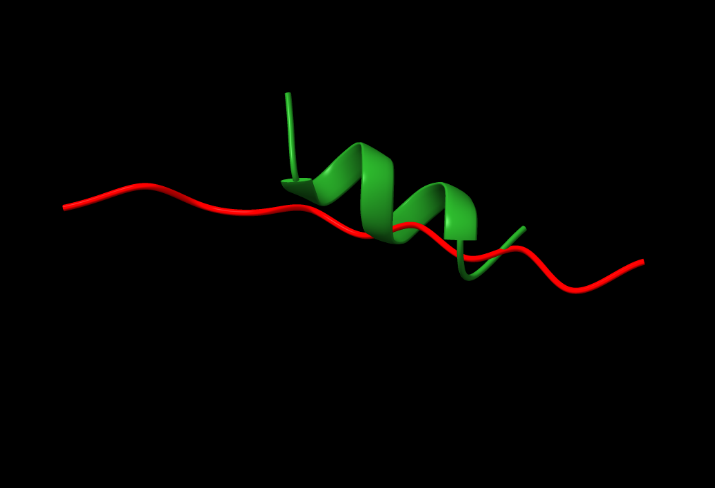

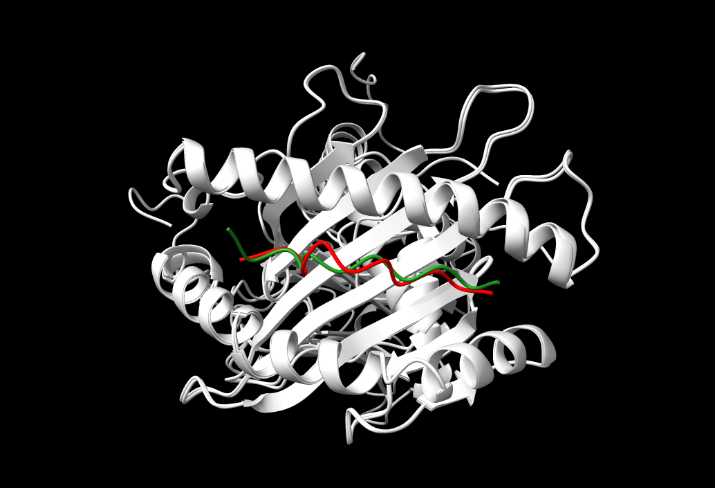
**

Epitope Pair 625331: Conformation of the pair epitopes, free (L) and bound (R) and corresponding Boltz-2 Confidence Scores for HHV-6 (red), T1DM (green), HHV-6/ HLA-B*38:02 (white), T1DM/ HLA-B*38:02 (white): 0.75, 0.74, 0.88, 0.88.


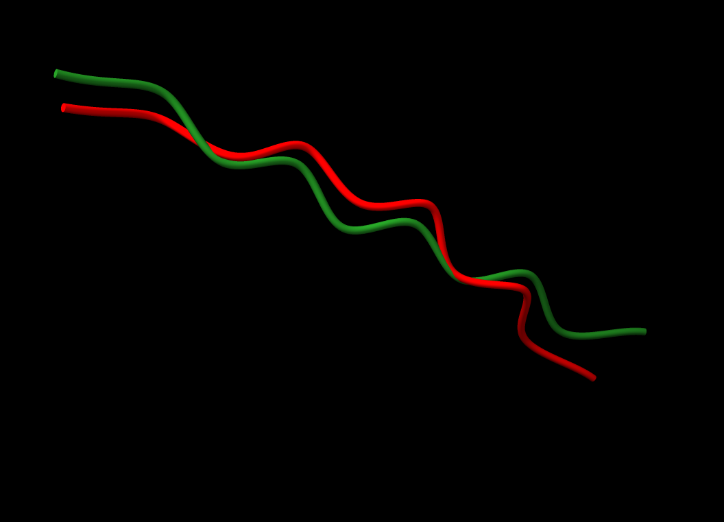
 **
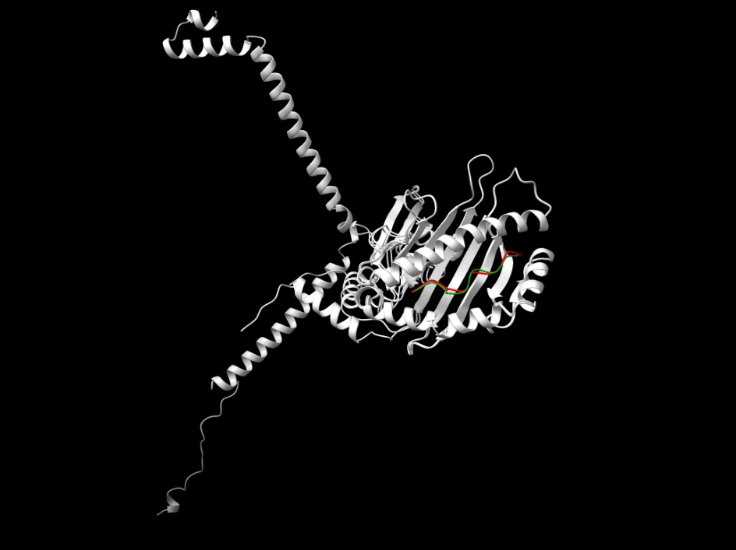
**

Epitope Pair 771155: Conformation of the pair epitopes, free and corresponding Boltz-2 Confidence Scores for HHV-6 (red), T1DM (green): (No HLA molecule): 0.73, 0.80.

**
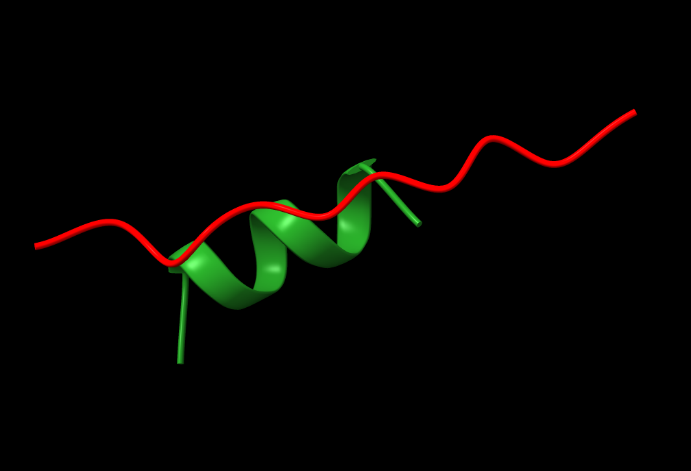
**

Epitope Pair 890415: Conformation of the pair epitopes, free (L) and bound (R) and corresponding Boltz-2 Confidence Scores for HHV-6 (red), T1DM (green), HHV-6/ HLA-A*25:01 (white), T1DM/ HLA-A*25:01 (white): 0.77, 0.83, 0.89, 0.89.

**
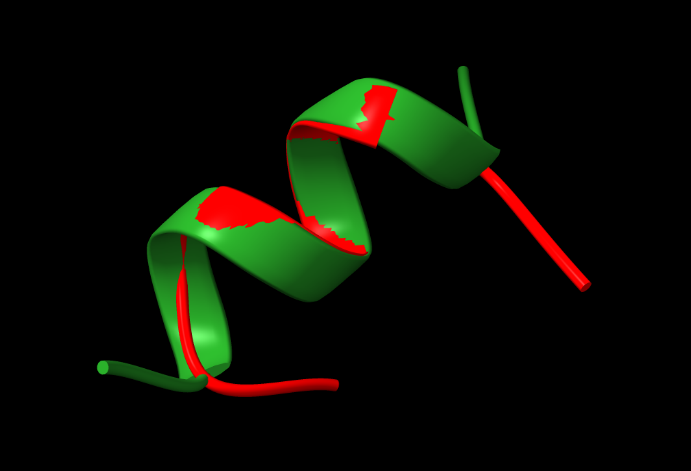

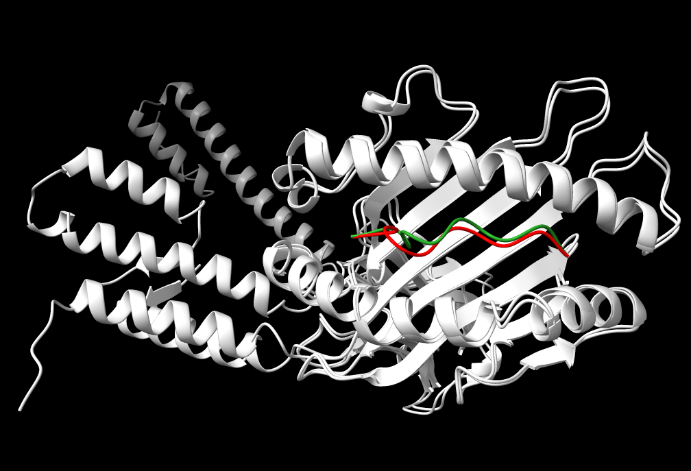
**

Epitope Pair 935014*:* Conformation of the pair epitopes, free (L) and bound (R) and corresponding Boltz-2 Confidence Scores for HHV-6 (red), T1DM (green), HHV-6/ HLA-A*25:01 (white), T1DM/ HLA-A*25:01 (white): 0.78, 0.84, 0.84, 0.87.

**
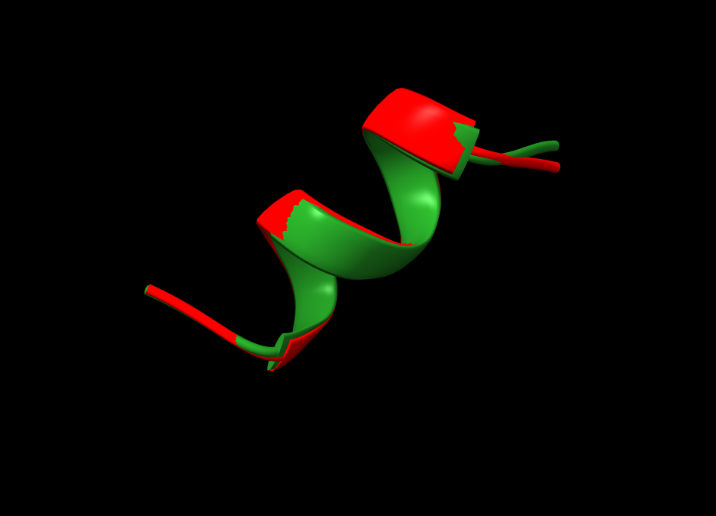

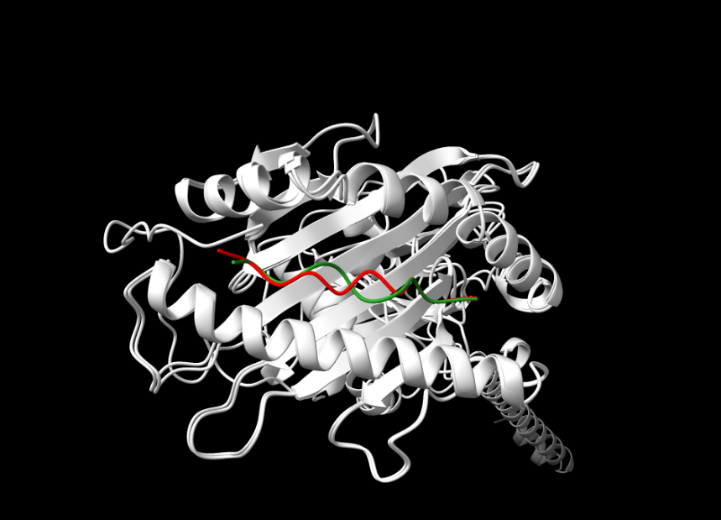
**

Epitope Pair 935131: Conformation of the pair epitopes, free (L) and bound (R) and corresponding Boltz-2 Confidence Scores for HHV-6 (red), T1DM (green), HHV-6/ HLA-A*25:01 (white), T1DM/ HLA-A*25:01 (white): 0.65, 0.76, 0.84, 0.86.

**
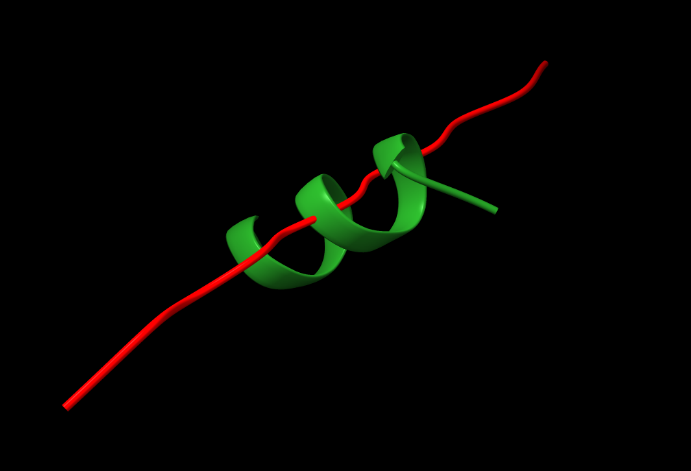

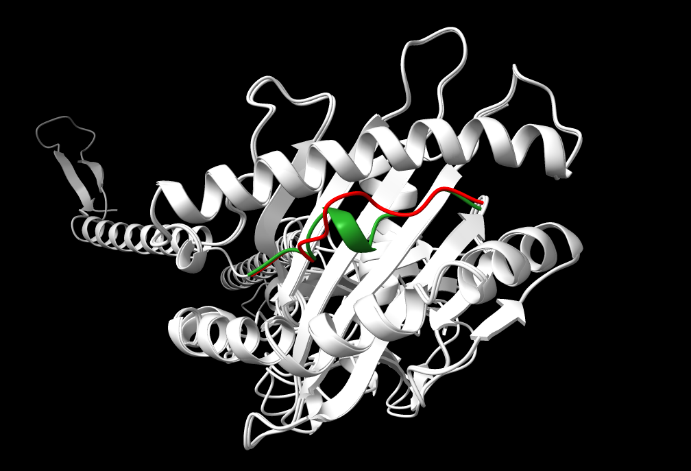
**

**Figure S4:** Electrostatic potentials of epitopes biding to HLAs molecules

Epitope pair 163076: HHV-6 (green), T1DM (green), HLA-A*25:01 electrostatic potential red for negative potential, blue for positive potential, and white for neutral regions.


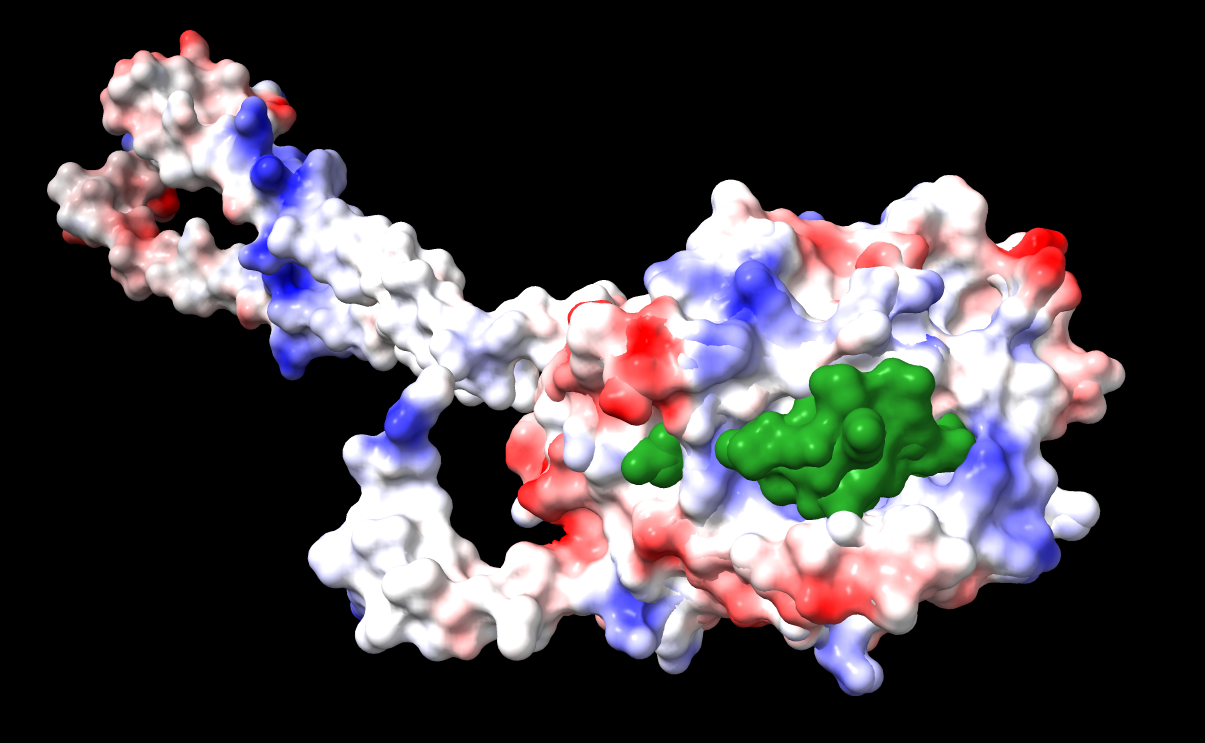


Epitope pair 185874: HHV-6 (green), T1DM (green), HLA-A*25:01 electrostatic potential red for negative potential, blue for positive potential, and white for neutral regions.

**
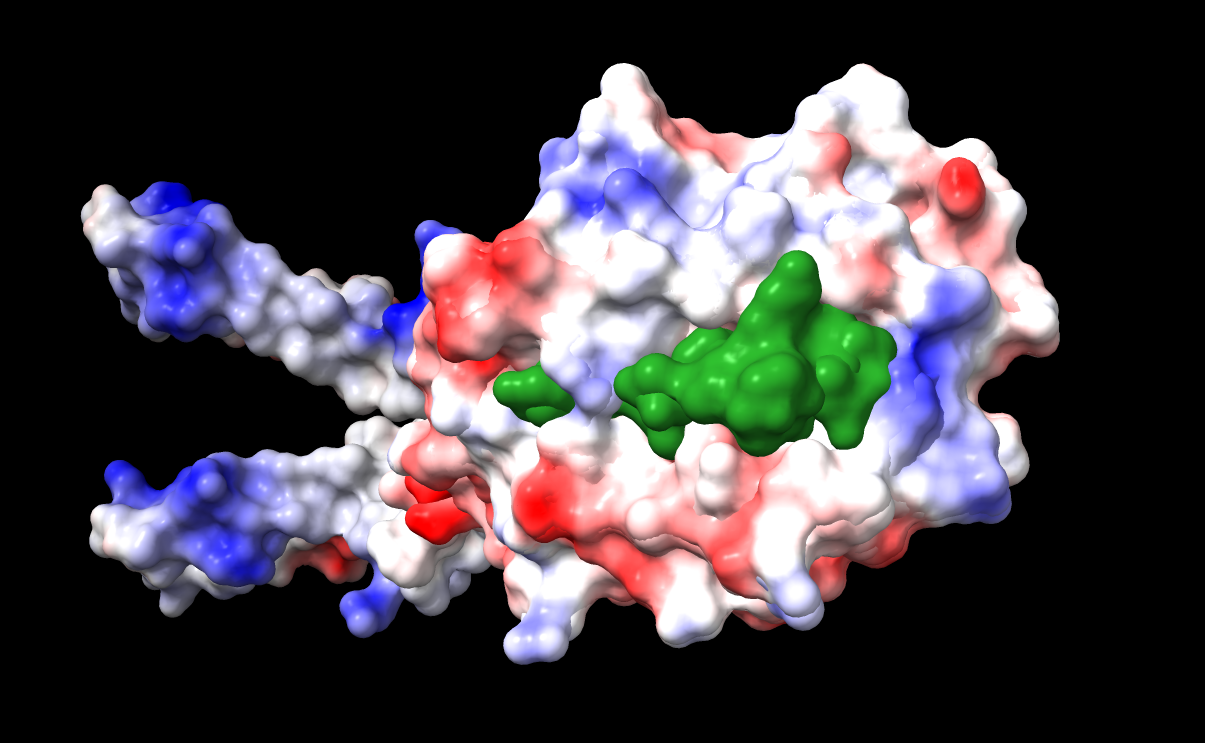
**

Epitope pair 430465: HHV-6 (green), T1DM (green), HLA-A*25:01 electrostatic potential red for negative potential, blue for positive potential, and white for neutral regions.


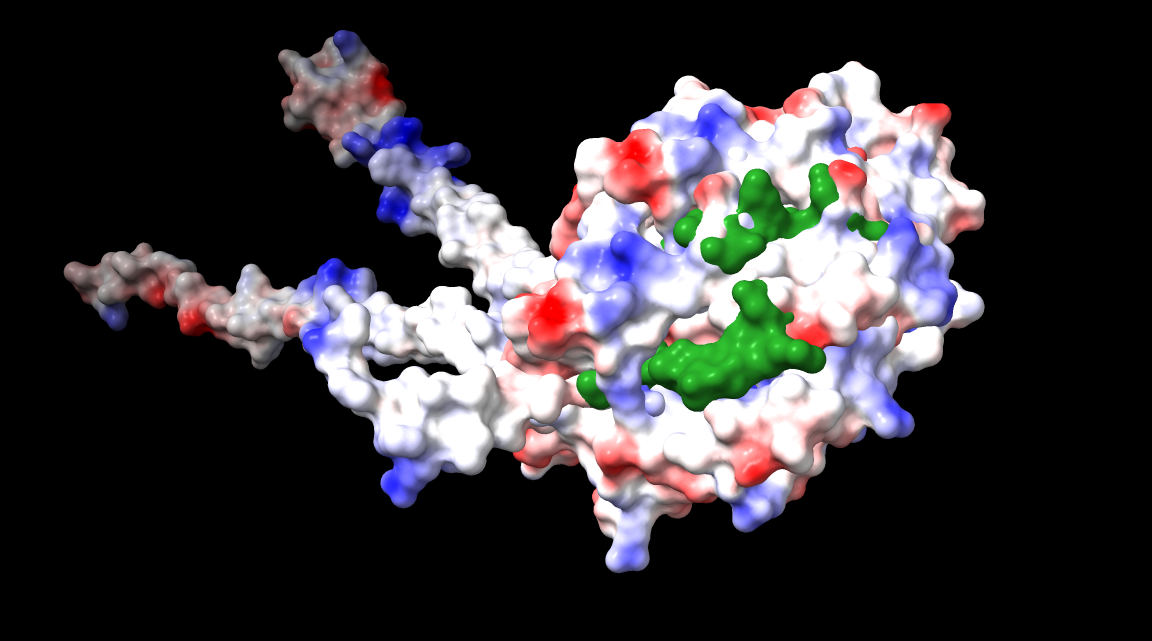


Epitope pair 431152: HHV-6 (green), T1DM (green), HLA-A*25:01 electrostatic potential red for negative potential, blue for positive potential, and white for neutral regions.


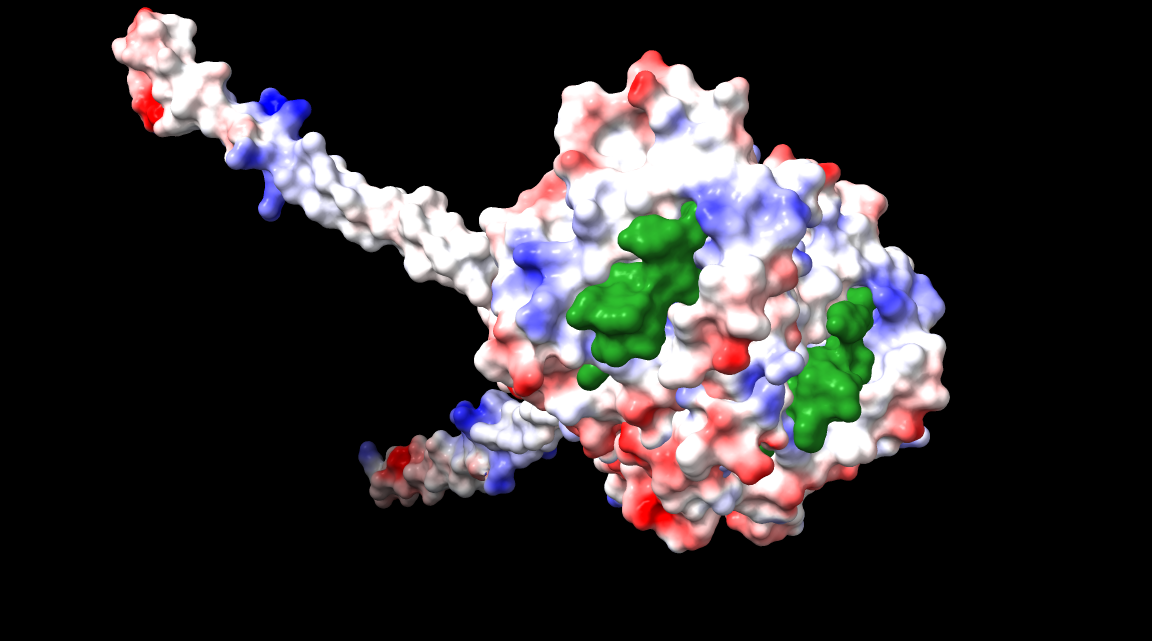


Epitope pair 431175: HHV-6 (green), T1DM (green), HLA-A*01:01 electrostatic potential red for negative potential, blue for positive potential, and white for neutral regions.


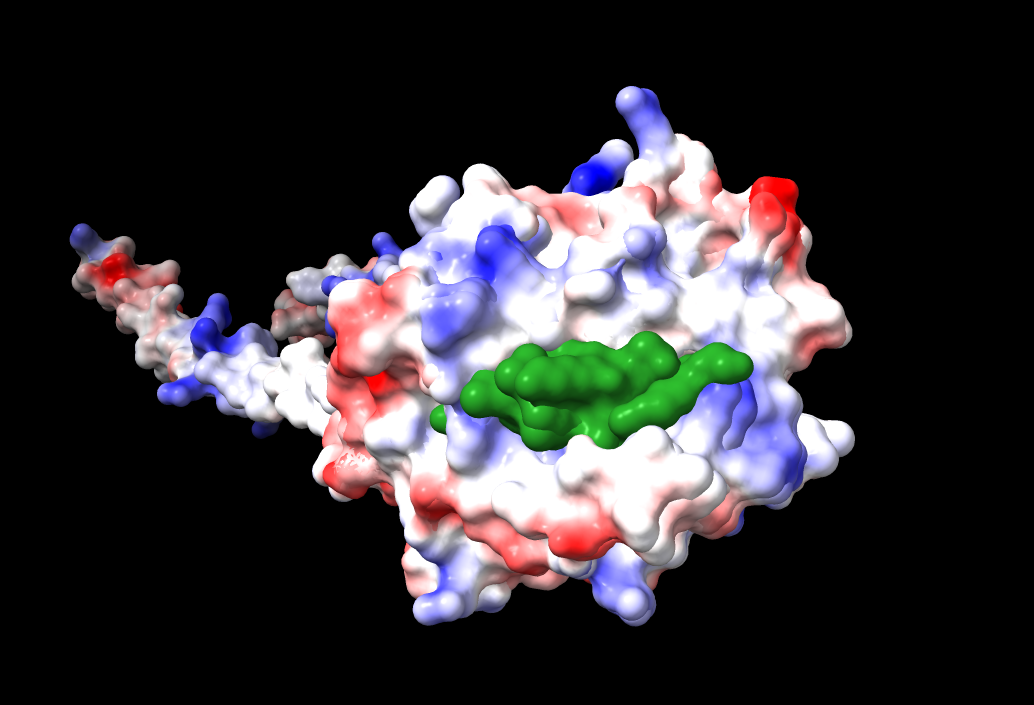


Epitope pair 431177: HHV-6 (green), T1DM (green), HLA-A*25:01 electrostatic potential red for negative potential, blue for positive potential, and white for neutral regions.


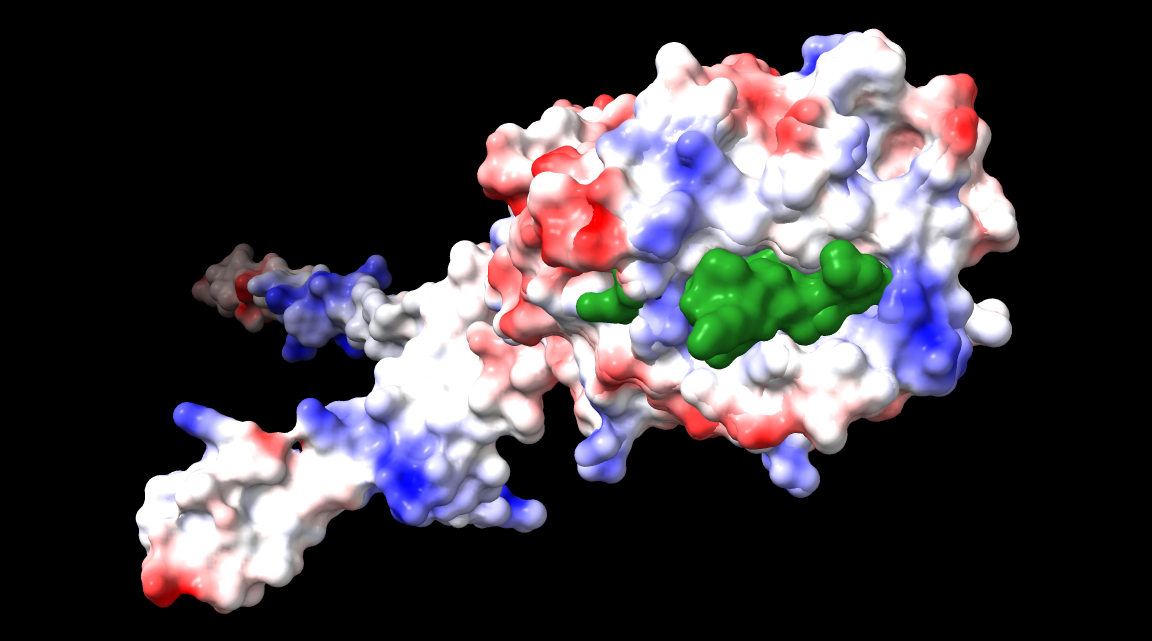


Epitope pair 431594: HHV-6 (green), T1DM (green), HLA-A*01:01 electrostatic potential red for negative potential, blue for positive potential, and white for neutral regions.


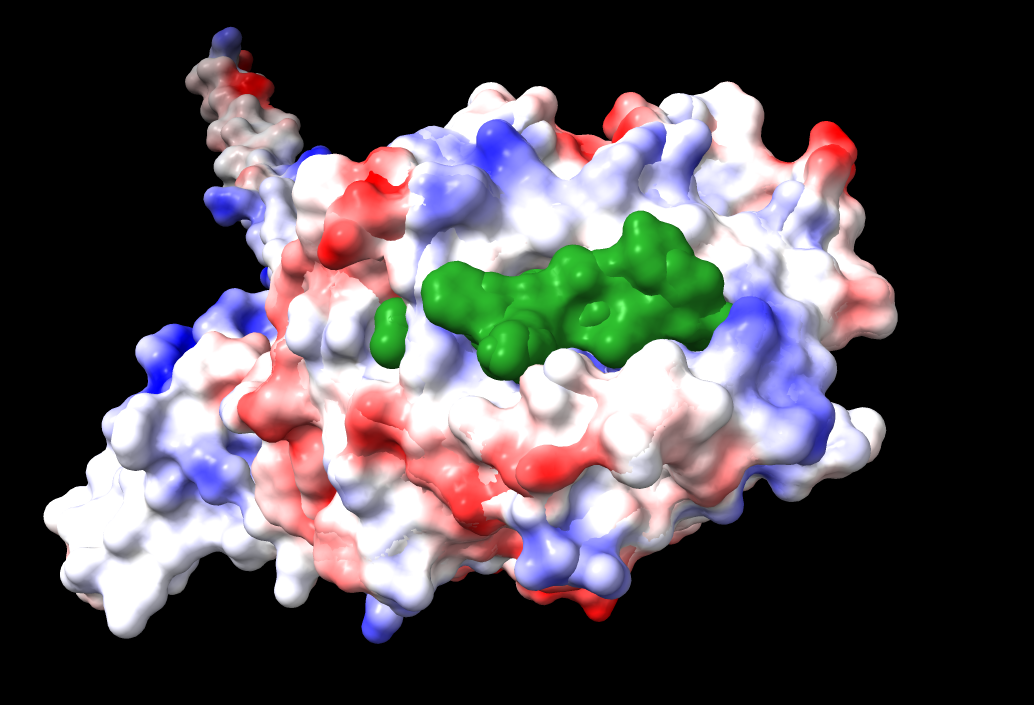


Epitope pair 448259: HHV-6 (green), T1DM (green), HLA-A*25:01 electrostatic potential red for negative potential, blue for positive potential, and white for neutral regions.


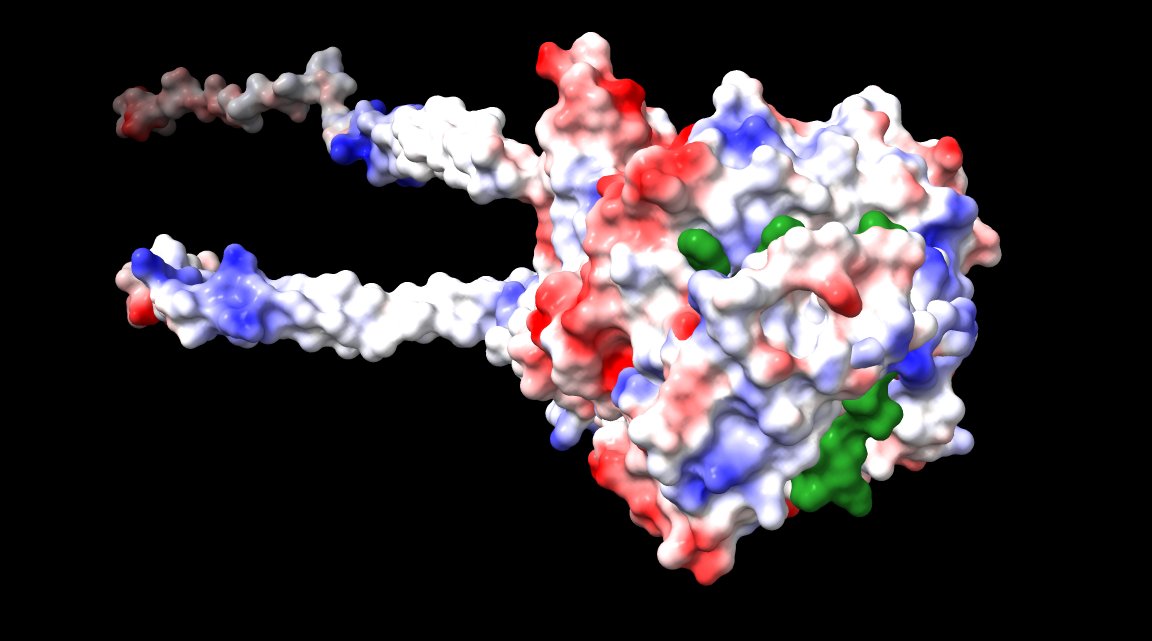


Epitope pair 449222: HHV-6 (green), T1DM (green), HLA-A*25:01 electrostatic potential red for negative potential, blue for positive potential, and white for neutral regions.

**
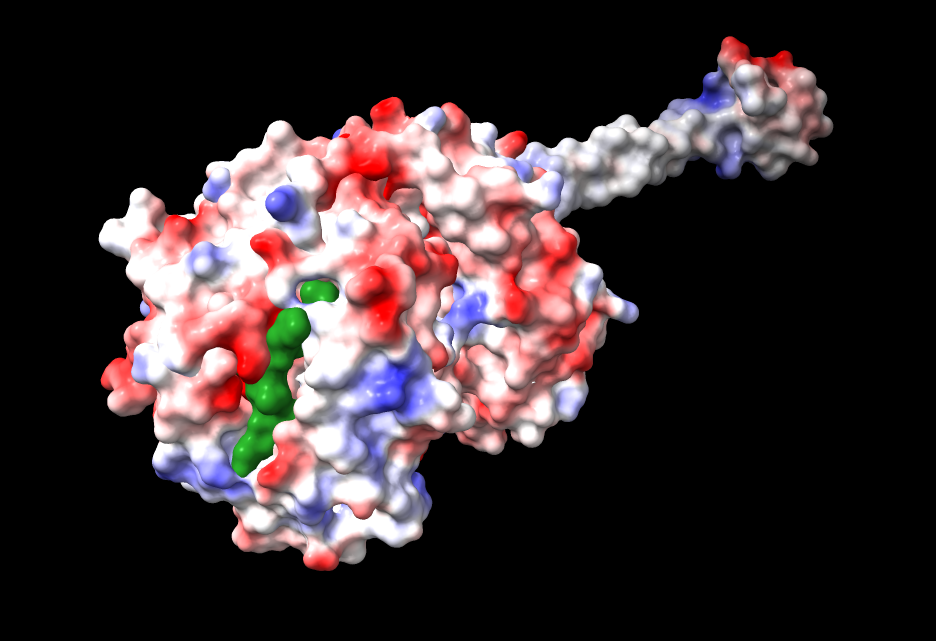
**

Epitope pair 468440: HHV-6 (green), T1DM (green), HLA-A*01:01 electrostatic potential red for negative potential, blue for positive potential, and white for neutral regions.

*
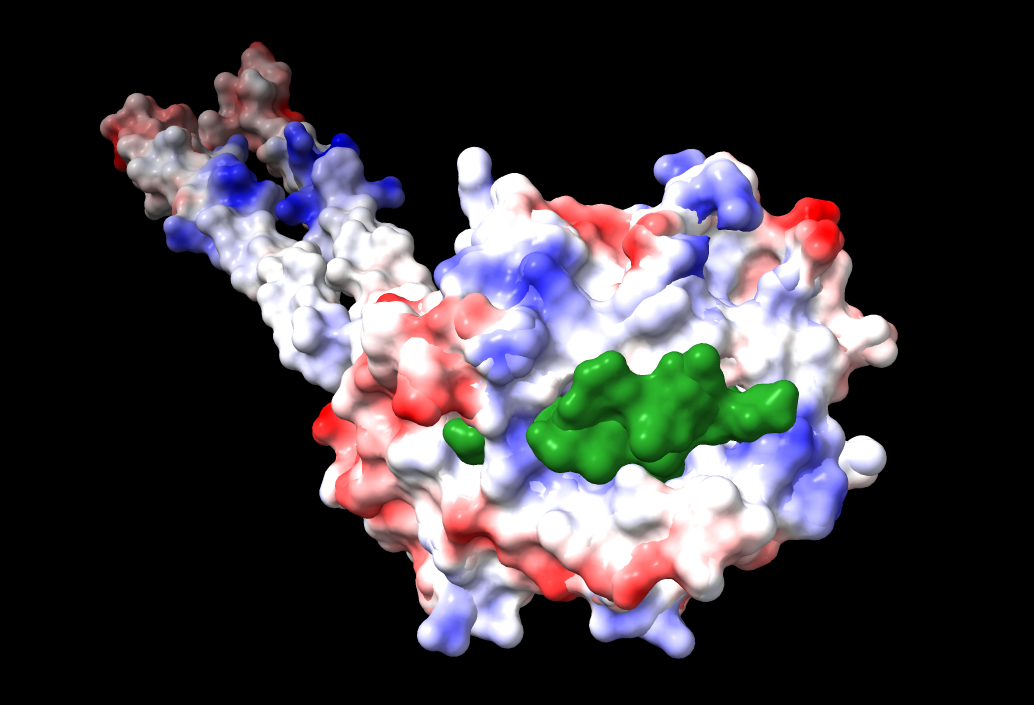
*

Epitope pair 482558: HHV-6 (green), T1DM (green), HLA-B*18:01 electrostatic potential red for negative potential, blue for positive potential, and white for neutral regions.

**
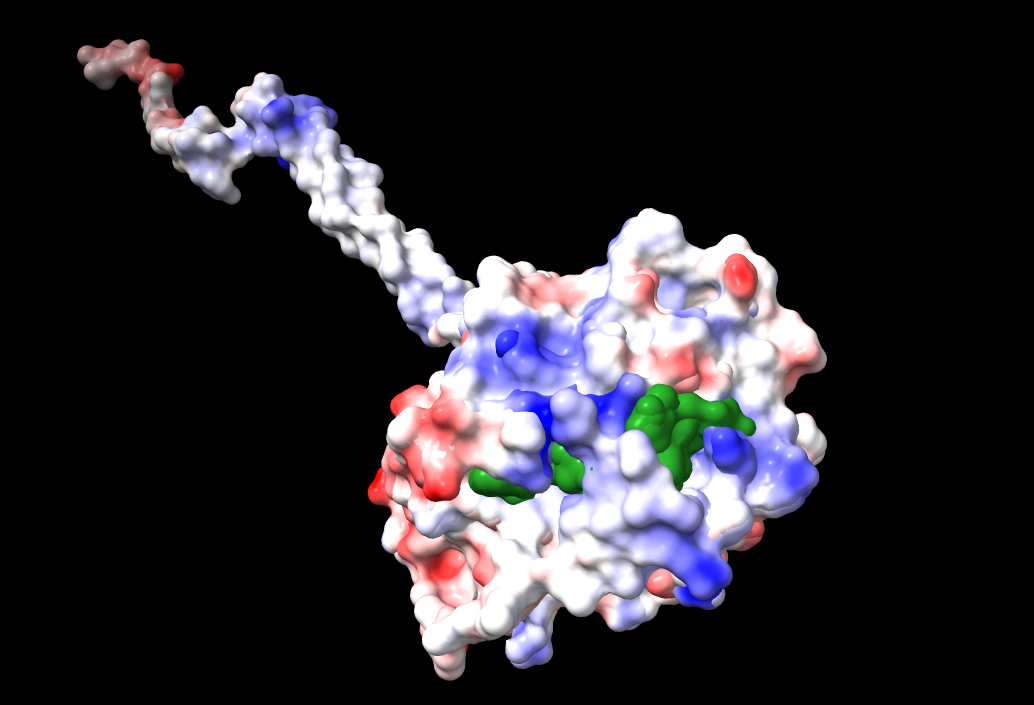
**

Epitope pair 541490: HHV-6 (green), T1DM (green), HLA-B*08:01 electrostatic potential red for negative potential, blue for positive potential, and white for neutral regions.

**
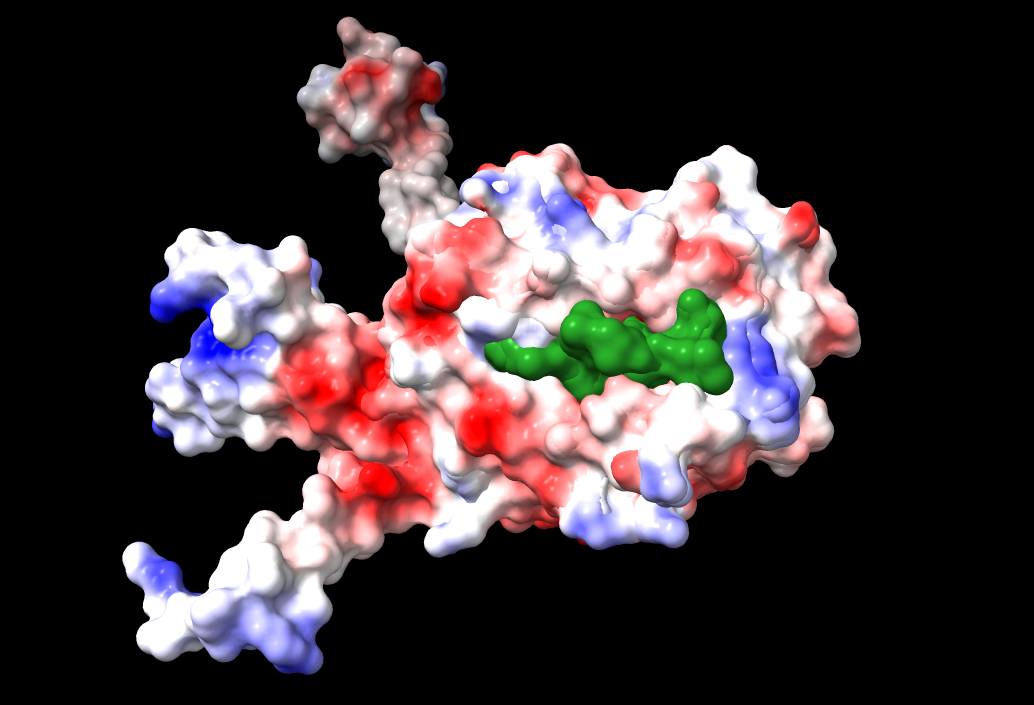
**

Epitope pair 562998: HHV-6 (green), T1DM (green), HLA-A*25:01 electrostatic potential red for negative potential, blue for positive potential, and white for neutral regions.

**
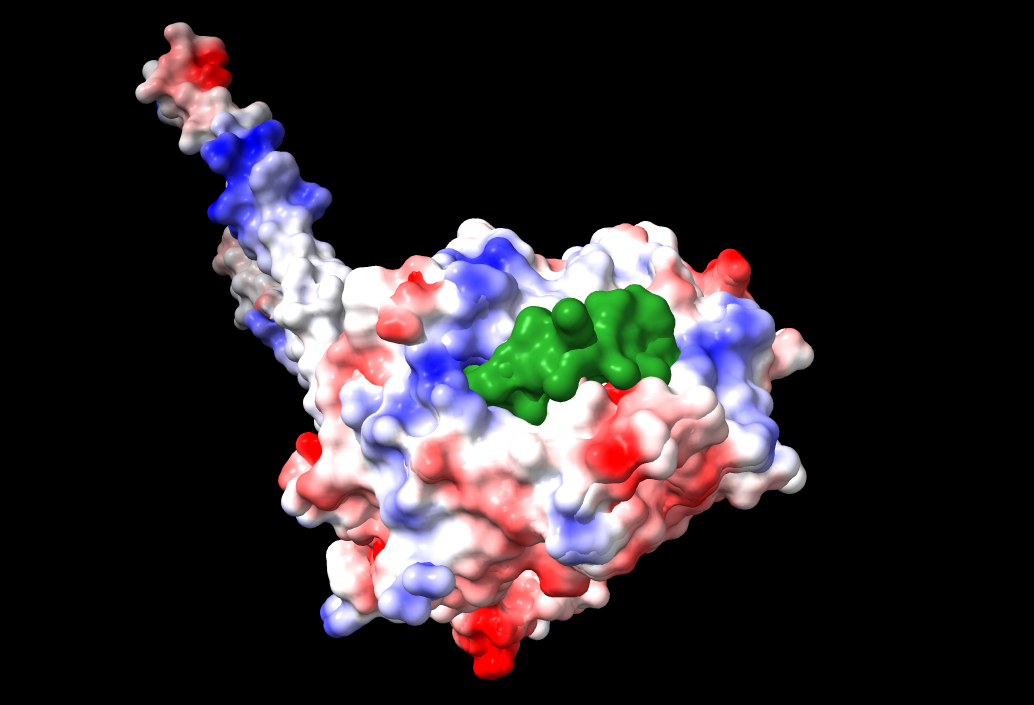
**

Epitope pair 563936: HHV-6 (green), T1DM (green), HLA-A*25:01 electrostatic potential red for negative potential, blue for positive potential, and white for neutral regions.


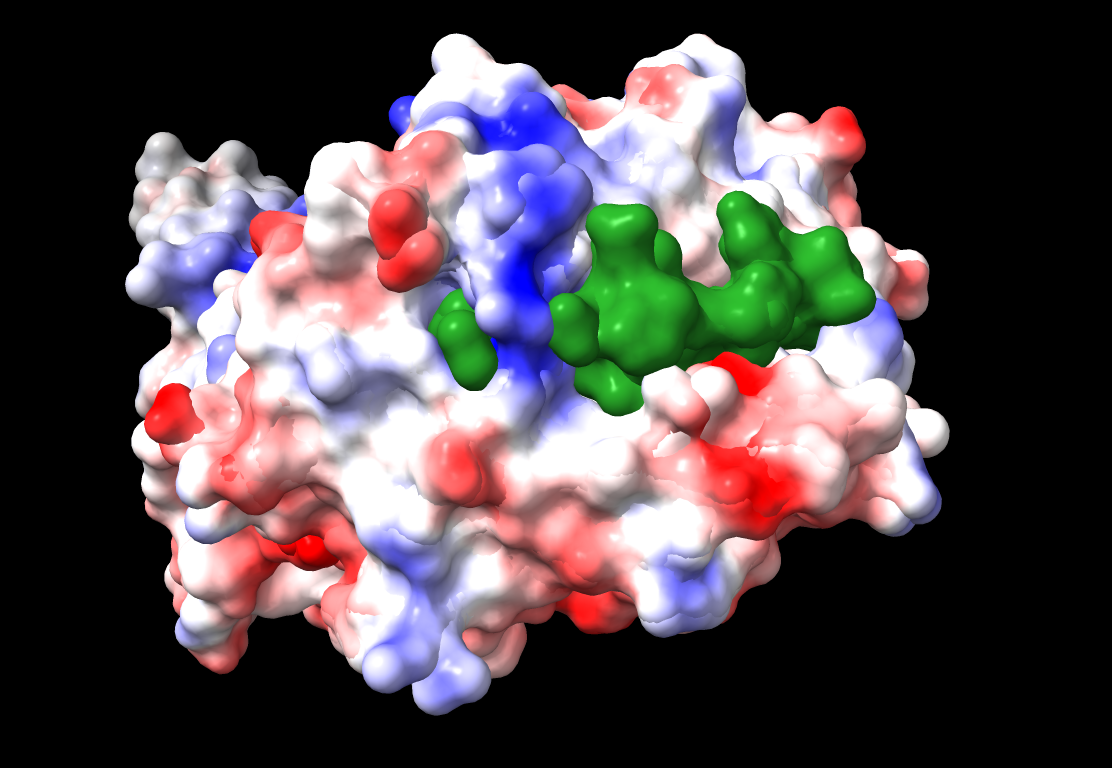


Epitope pair 571479**:** HHV-6 (green), T1DM (green), HLA-A*08:01 electrostatic potential red for negative potential, blue for positive potential, and white for neutral regions.

**
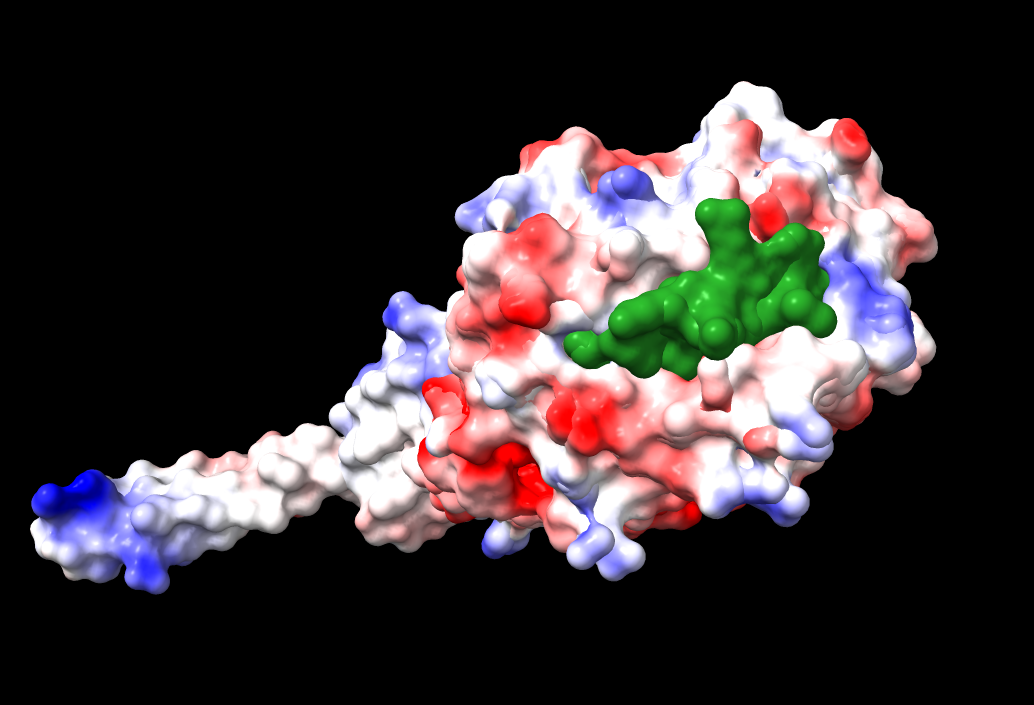
**

Epitope pair 571480: HHV-6 (green), T1DM (green), HLA-A*25:01 electrostatic potential red for negative potential, blue for positive potential, and white for neutral regions.

**
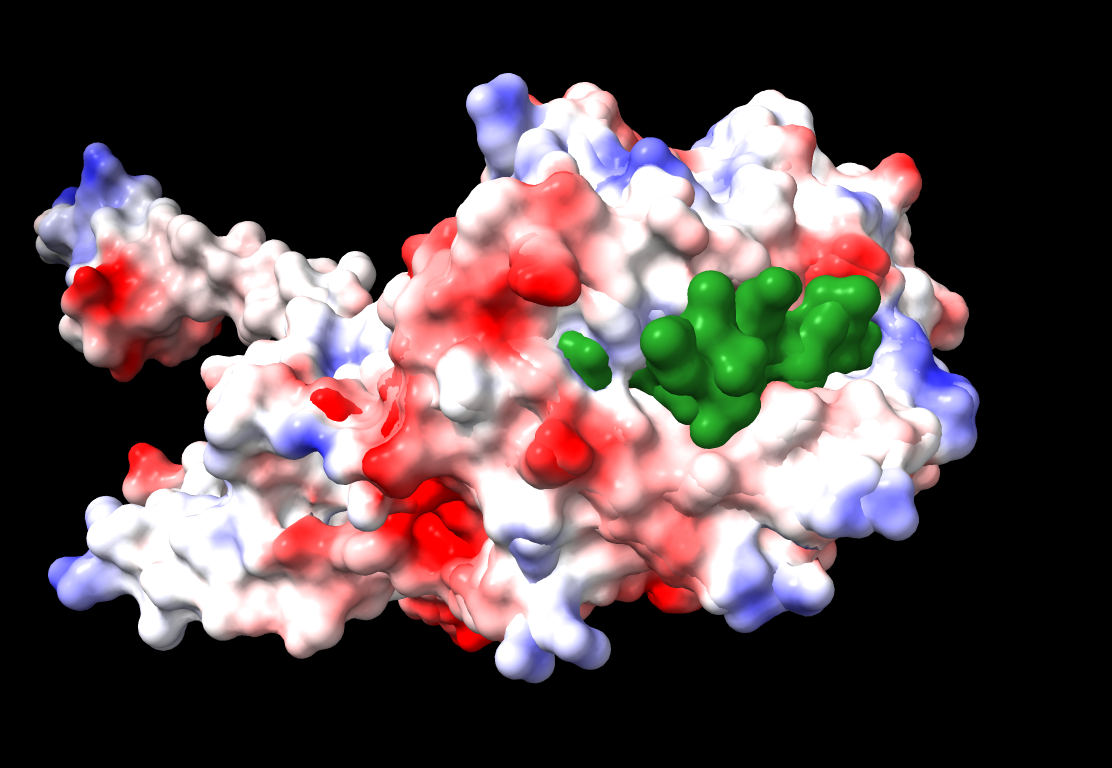
**

Epitope pair 573185: HHV-6 (green), T1DM (green), HLA-A*25:01 electrostatic potential red for negative potential, blue for positive potential, and white for neutral regions.

**
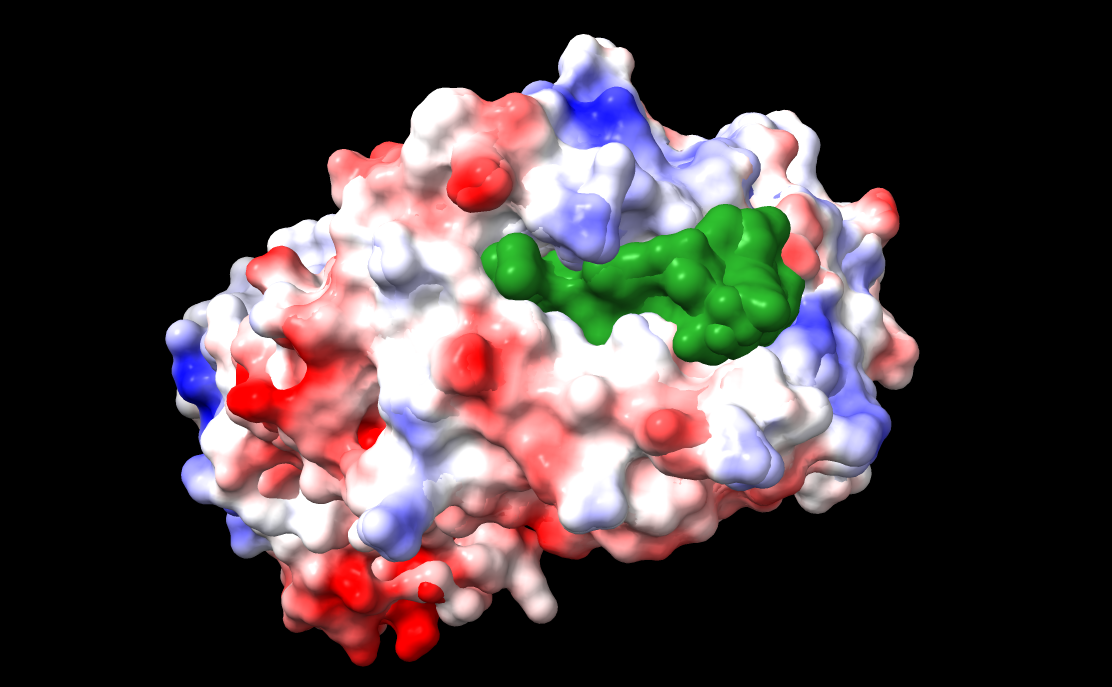
**

Epitope pair 602576: HHV-6 (green), T1DM (green), HLA-A*25:01 electrostatic potential red for negative potential, blue for positive potential, and white for neutral regions.


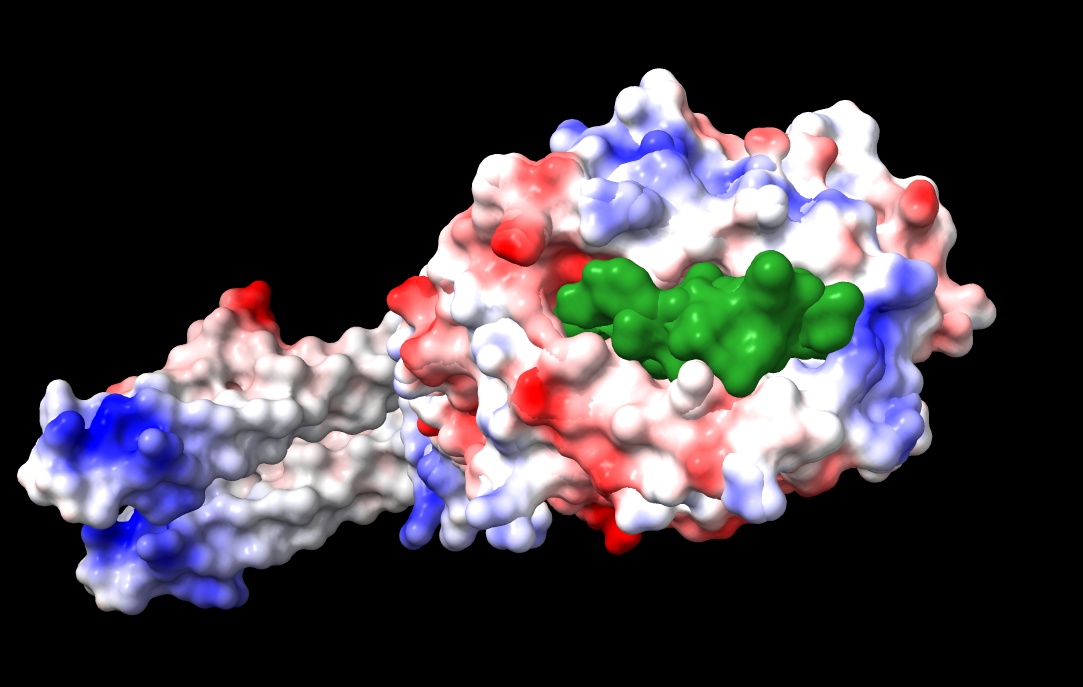


Epitope pair 620896: HHV-6 (green), T1DM (green), HLA-A*23:01 electrostatic potential red for negative potential, blue for positive potential, and white for neutral regions.

**
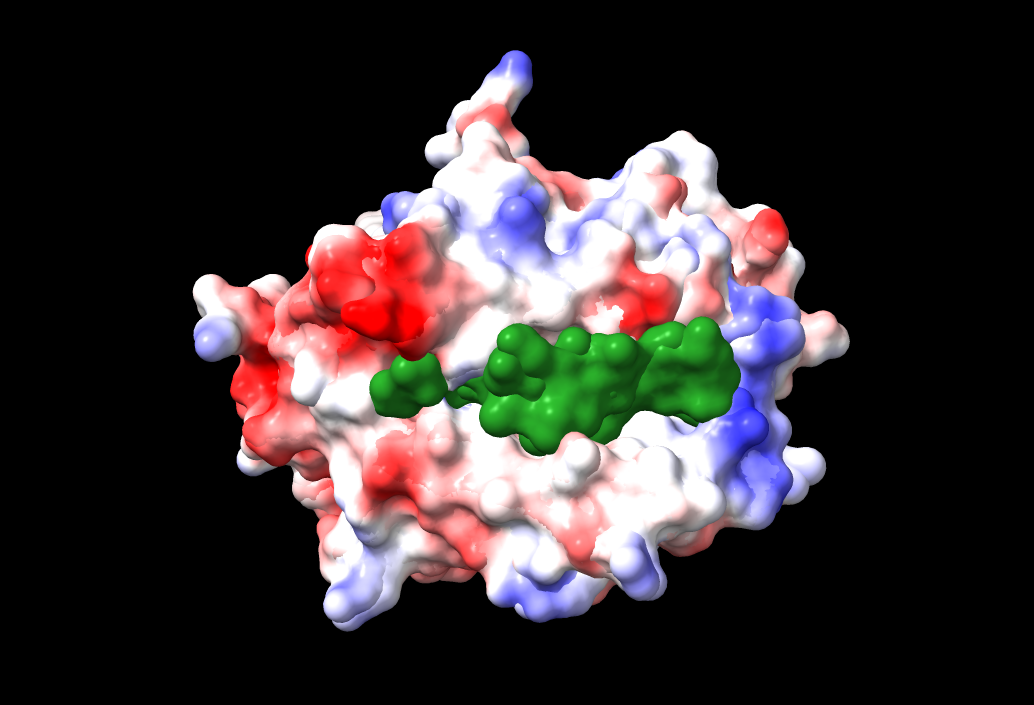
**

Epitope pair 625331: HHV-6 (green), T1DM (green), HLA-B*38:02 electrostatic potential red for negative potential, blue for positive potential, and white for neutral regions.

**
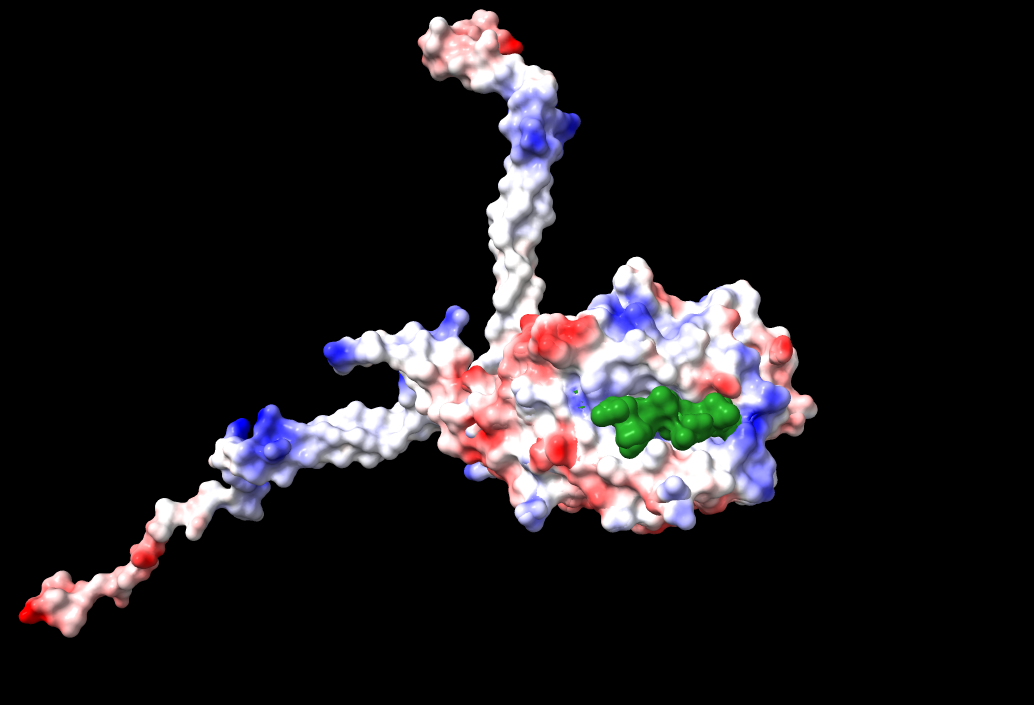
**

Epitope pair 890415: HHV-6 (green), T1DM (green), HLA-A*25:01 electrostatic potential red for negative potential, blue for positive potential, and white for neutral regions.

**
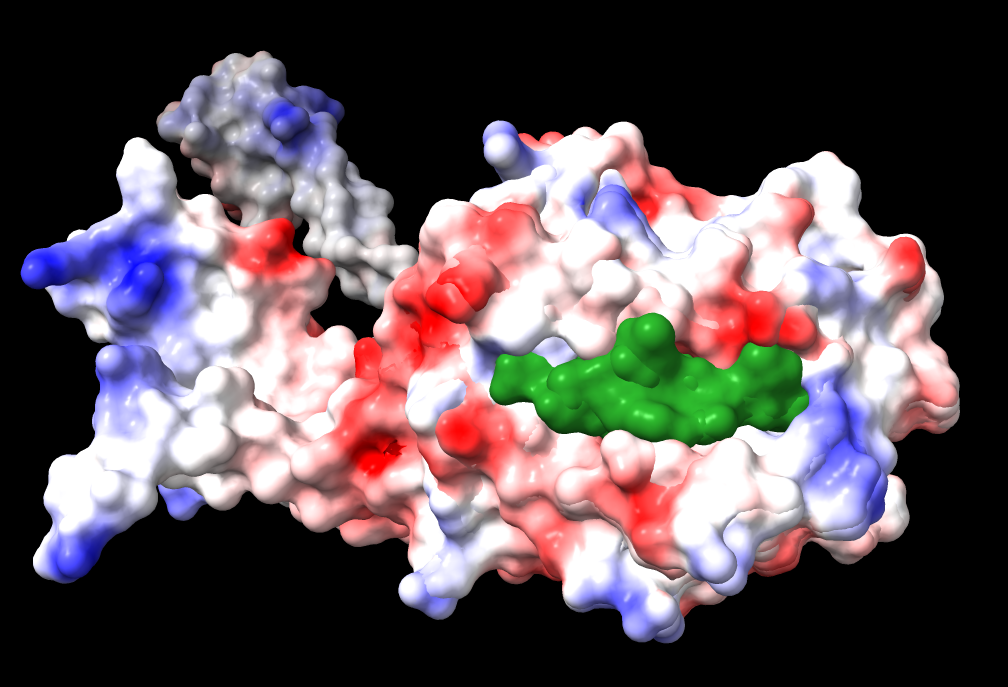
**

Epitope pair 935014: HHV-6 (green), T1DM (green), HLA-A*25:01 electrostatic potential red for negative potential, blue for positive potential, and white for neutral regions.

**
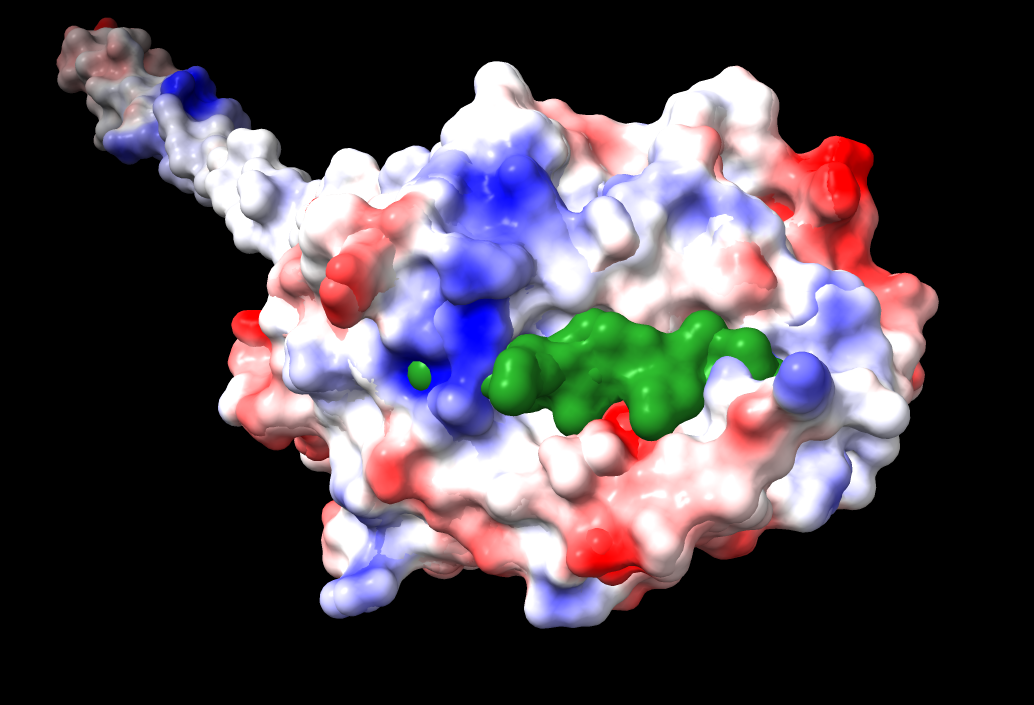
**

Epitope pair 935131: HHV-6 (green), T1DM (green), HLA-A*25:01 electrostatic potential red for negative potential, blue for positive potential, and white for neutral regions.


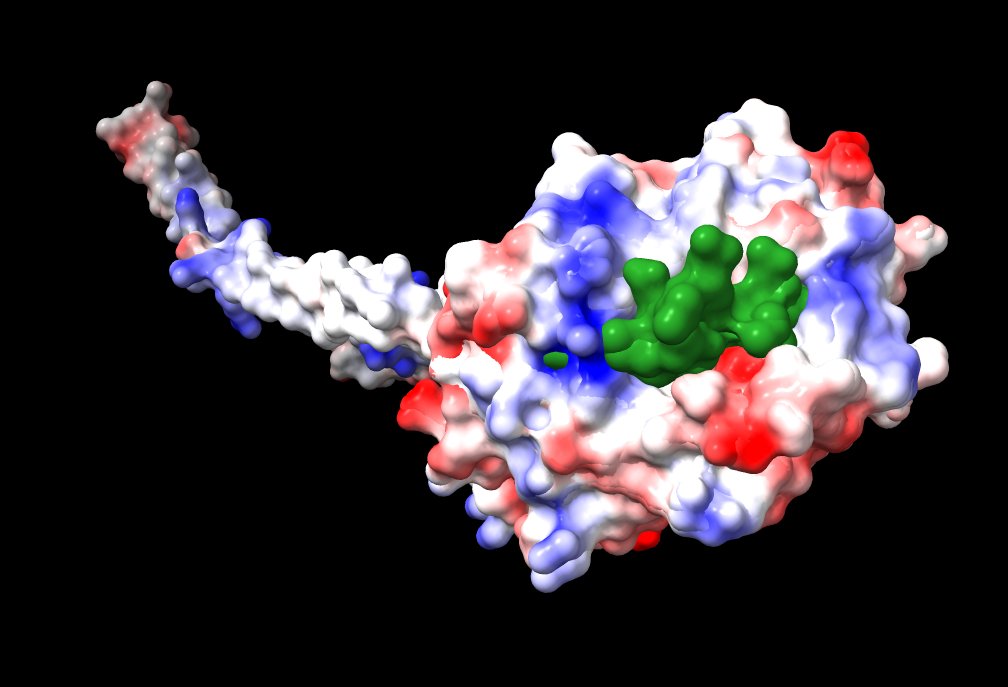

Supplement: iqag011_Supplementary_Data [file iqag011_supplementary_data.docx]
